# Mapping Technologies, Support Functions, Evaluation Outcomes, and Safety Governance Related to Conversational AI Agents for Psychological Support of Patients With Cancer: Scoping Review

**DOI:** 10.2196/105599

**Published:** 2026-10-19

**Authors:** Zhiying Shen, Shuangjiao Shi, Lian Mao, Chengyuan Li, Jianfei Xie

**Affiliations:** 1 Department of Hematology Third Xiangya Hospital Central South University Changsha, Hunan China; 2 Nursing Department Third Xiangya Hospital Central South University Changsha, Hunan China

**Keywords:** conversational artificial intelligence, conversational AI, chatbot, cancer, psychological support, psycho-oncology, digital health, scoping review

## Abstract

**Background:**

Psychological distress and unmet psychosocial needs are common across the cancer continuum. Conversational AI agents may offer scalable support, but their technologies, support functions, evaluation approaches, and safety governance remain fragmented.

**Objective:**

This review aimed to map the technologies, psychological support functions, evaluation outcomes, and safety governance of conversational AI used by patients with cancer and cancer survivors.

**Methods:**

We conducted a scoping review following Joanna Briggs Institute (JBI) methodology and the PRISMA-ScR (Preferred Reporting Items for Systematic Reviews and Meta-Analyses Extension for Scoping Reviews). Thirteen databases and additional registers were searched through May 5, 2026. Eligible reports examined conversational agents in real or simulated patient-facing oncology contexts with explicit psychological or psychosocial relevance. We summarized system forms and support functions by project and other characteristics by report. No formal critical appraisal was undertaken.

**Results:**

We included 24 reports representing 25 study units and 23 underlying projects. Large language model (LLM) or generative AI systems accounted for 39.1% (9/23) of projects. Information or education (23/23, 100%) and symptom or self-management support (n=20, 87%) were the most frequently mapped functions, although these counts included delivered, planned, simulated, co-designed, and participant-reported functions. Structured psychological or behavioral components were delivered in 21.7% (5/23) of projects. An explicit theoretical or conceptual framework was reported in 25% (6/24) of reports. Reports or plans to assess acceptability or user experience (17/24, 70.8%) and engagement (13/24, 54.2%) were more common than reports or plans to assess psychosocial clinical outcomes (8/24, 33.3%) or safety and adverse events (4/24, 16.7%). Human or expert oversight was reported in 62.5% (15/24) of reports, crisis escalation or referral in 33.3% (8/24) of reports, and user-facing AI identity disclosure in 12.5% (3/24) of reports.

**Conclusions:**

Conversational AI research in psycho-oncology spans heterogeneous technologies and stages of development. Clinical and safety evaluation, theoretical foundations, population representation, and oversight responsibilities remain incompletely reported. These findings represent a map of reporting patterns and do not establish effectiveness or safety. Future studies should evaluate psychosocial benefit and safety prospectively and define professional oversight and referral responsibilities.

**Trial Registration:**

OSF Registries UM65W; https://osf.io/um65w

## Introduction

Cancer creates substantial needs for supportive care alongside diagnosis and treatment. The Global Burden of Disease Study 2023 estimated 18.5 million new cancer cases and 10.4 million cancer deaths globally in 2023 [1]. Uncertainty, fear of recurrence or progression, symptoms, functional disruption, and financial strain can contribute to distress, anxiety, depression, sleep disturbance, and unmet psychosocial needs [2,3]. An umbrella review reported pooled prevalence estimates of 33.16% for depression and 30.55% for anxiety among patients with cancer [4]. Accessible psychological support is therefore an important component of cancer care.

Digital interventions may improve mental health outcomes among patients with cancer and cancer survivors [5], but evidence across digital modalities does not fully characterize conversational systems. Their dialogue mechanisms, support functions, therapeutic foundations, and oversight require specific examination. This is relevant in settings in which resource limitations, stigma, and organizational barriers restrict access to psychosocial assessment and referral [6-8].

Conversational AI includes text chatbots, voice assistants, social robots, and large language model (LLM)–based agents. A systematic review and meta-analysis of 17 studies involving 1817 participants examined conversational agents across cancer care and identified heterogeneity in interventions, outcomes, and implementation [9]. Individual studies have investigated positive psychology support after cancer treatment [10], generative AI support for pediatric and young adult patients [11], and ChatGPT-based counseling before chemotherapy [12]. A focused synthesis is needed to characterize their psychological support functions and conditions of use.

Related reviews have examined health care chatbots [13] and LLMs for cancer decision-making [14], while empirical evaluations have considered response quality, empathy, and unsafe answers [15-17]. Kılınç and Oflaz [18] synthesized 7 English-language randomized trials of AI-based psychosocial interventions in cancer, including conversational and nonconversational tools, and assessed outcomes and risk of bias. Their review also discussed therapeutic foundations and ethical concerns. The present review complements that synthesis by focusing on conversational systems across trial and nontrial evidence and systematically mapping support functions, theoretical foundations, population representation, professional oversight, and safety governance.

This scoping review aimed to map the technologies, psychological support functions, evaluation domains, and safety governance of conversational AI for patients with cancer and cancer survivors. The heterogeneous technologies, study designs, and stages of development warranted an evidence-mapping approach.

## Methods

### Design

This scoping review was conducted in accordance with Joanna Briggs Institute (JBI) methodology [19] and reported according to the PRISMA-ScR (Preferred Reporting Items for Systematic Reviews and Meta-Analyses Extension for Scoping Reviews) checklist [20]. The review addressed 3 questions: (1) What technologies and support functions have been reported in conversational AI agents used for psychological support among patients with cancer? (2) What evaluation outcomes have been used to assess these agents? (3) How has safety governance been addressed?

### Protocol and Registration

The protocol was prospectively registered on the OSF before formal title and abstract screening and data charting (UM65W). Any amendments to the registered protocol were documented on OSF, including the date, description, and rationale for each change.

### Eligibility Criteria

Eligibility criteria were structured according to the population, concept, and context framework. Studies were included if they involved patients with cancer or cancer survivors or used explicitly patient-facing, simulated oncology scenarios and examined AI-supported systems capable of interactive text, voice, or multimodal dialogue. The conversational function had to be relevant to psychological, emotional, psychosocial, coping-related, survivorship-related, supportive care, or quality-of-life support. Treatment engagement, adherence, symptom management, or self-management was eligible only when the report explicitly linked it to psychological or emotional support, coping, patient activation, supportive communication, or a structured psychological or behavioral strategy. Eligibility was determined from the reported study context and content rather than from the technology label alone.

Original empirical studies using quantitative, qualitative, or mixed methods designs were considered. Full peer-reviewed conference papers were included when sufficient methodological and outcome information was available. Published protocols and trial registry records were included only when they provided sufficient information on the conversational AI agent, psychological support content, or planned evaluation. Both purpose-built conversational agents and general-purpose LLMs were eligible, but a general-purpose model was included only when the report explicitly evaluated its use in a defined oncology support context involving real patient questions, patient-derived needs, clearly constructed simulated patient scenarios, or patient-facing psychological or supportive care content. Reports based solely on frequently asked questions on public websites, general health information, or expert-generated questions without a real or simulated patient-facing context were excluded. Evidence about independent, unsupervised, real-world use of general-purpose LLMs by patients was included only if it was itself examined in an eligible study; such use was not inferred from evaluations of model-generated responses. No language restrictions were applied. The detailed inclusion and exclusion criteria are presented in Table 1.

**Table 1:** Inclusion and exclusion criteria.

Characteristics	Inclusion criteria	Exclusion criteria
Population	Patients with cancer or cancer survivors at any stage of the cancer continuum, including diagnosis, treatment, follow-up, survivorship, supportive care, or palliative care	No patient or cancer survivor population, or cancer-specific patient data or content could be separated in mixed-population studies
Conversational AI agent	AI-supported conversational systems providing text, voice, or multimodal patient-facing or simulated patient-facing responses, including chatbots, virtual agents, voice assistants, social robots, large language model or generative AI agents, and interactive question-and-answer systems	Non-AI digital tools, static websites or apps, reminders, scheduling tools, or systems without an AI-supported dialogue mechanism
Psychological support relevance	Psychological, emotional, psychosocial, coping, survivorship, supportive care, or quality-of-life relevance. Eligible topics included distress, anxiety or depression, coping, self-efficacy, fear of recurrence or progression, body image, fertility, intimacy, communication, sleep-related concerns, and structured strategies such as cognitive behavioral therapy, mindfulness, relaxation, problem-solving, positive psychology, or psychoeducation.	Only symptom collection, toxicity alerts, medication or appointment reminders, logistics, general cancer information, genetic testing navigation, or administrative functions without psychological, emotional, coping-related, treatment engagement, survivorship, or quality-of-life support content, mechanisms, outcomes, or patient-facing scenarios
Context	Oncology-related real-world or simulated patient-facing contexts across cancer care, survivorship, supportive care, palliative care, psycho-oncology care, digital health, community or home-based care, or nurse-supported settings	Cancer used only as a technical, diagnostic, educational, or general information example without a patient-facing or simulated patient-facing oncology, supportive care, or psychological support context
Types of evidence sources	Original quantitative, qualitative, or mixed methods studies; development or evaluation studies; implementation studies; usability or acceptability studies; human-AI interaction studies; safety or governance studies; full peer-reviewed conference papers with sufficient methodological and outcome information; and protocols or registry records with sufficient planned study information	No extractable empirical data or insufficient planned study information relevant to the review questions

### Information Sources and Search Strategy

The following databases were searched from inception to May 5, 2026: PubMed, Embase, CINAHL, PsycInfo, Web of Science Core Collection, Scopus, Cochrane CENTRAL, IEEE Xplore, ACM Digital Library, CNKI, Wanfang Data, SinoMed, and VIP Database. Additional sources included ClinicalTrials.gov, the World Health Organization (WHO) International Clinical Trials Registry Platform, OSF Registries, reference lists of included reports and relevant reviews, and forward citation tracking of key included studies.

The search strategy combined 3 concept groups: AI-based conversational agents, cancer, and psychological support-related terms. The strategy was developed in PubMed and adapted for other databases according to controlled vocabulary, field tags, and search syntax. A focused search combining all 3 concept groups was conducted. A sensitivity search combining AI-based conversational agent terms and cancer terms was also performed to identify reports that might not explicitly mention psychological support in the titles or abstracts. Because terminology in conversational AI and LLMs evolves rapidly, both generic terms and selected model-specific terms were used. Detailed search strategies are presented in Multimedia Appendix 1.

### Selection of Sources of Evidence

Records were imported into Zotero (Corporation for Digital Scholarship) and deduplicated using its Duplicate Items function, with bibliographic details checked as needed. The deduplicated records were retained for screening.

Before formal screening, 2 reviewers independently pilot-screened approximately 30-50 titles and abstracts to test the eligibility criteria and clarify operational definitions. Borderline records, including symptom management chatbots, information support tools, genetic counseling agents, LLM response evaluation studies, rule-based or unclear AI systems, non-AI digital interventions, and mixed-population studies, were discussed to refine the screening guidance.

Two reviewers then independently screened titles and abstracts in Zotero using predefined tags and collections. Records judged eligible or potentially eligible by either reviewer proceeded to full-text screening. Full texts were assessed independently by 2 reviewers against the eligibility criteria. Disagreements at either screening stage were resolved through discussion, and unresolved disagreements were adjudicated by a third reviewer. Reasons for exclusion at the full-text stage were recorded and summarized in the PRISMA (Preferred Reporting Items for Systematic Reviews and Meta-Analyses) flow diagram.

Reference lists of included reports and relevant reviews were screened, and additional records underwent the same selection process. Reports from the same underlying project were linked for project-level analyses.

### Data Charting Process and Data Items

A standardized data charting form was developed in Microsoft Excel (version 16.0, Microsoft Corp) based on the review questions and a 4-domain framework: technologies, support functions, evaluation outcomes, and safety governance. Before full data charting, the form was pilot-tested on 5-10 included reports representing different evidence types, including intervention, development or evaluation, usability, LLM response evaluation, qualitative or co-design, and protocol reports. The form and coding guidance were refined iteratively until reviewers reached a shared understanding of the data elements and decision rules.

Two reviewers independently charted data from each included source using the finalized form. Discrepancies were resolved through discussion, and a third reviewer was consulted when consensus could not be reached. Information not explicitly described in the report was coded as “not reported”; information that was present but could not be classified reliably was coded as “unclear.” Neither code was interpreted as evidence that a system feature or population characteristic was absent. Each charted item retained a source locator (page, section, table, or quoted text, where available) to support auditability.

Charted data included bibliographic information, study design, population and cancer context, characteristics of the conversational AI agent, psychological support functions, evaluation outcomes, and reported safety, ethics, and governance features. Technology-related items included AI or system form, purpose-built vs general-purpose status, delivery platform, interaction modality, response generation mechanism, automation, personalization, and care integration. Population items included age and explicit representation of older adults or pediatric, adolescent, and young adult populations; cancer type and stage, including advanced or metastatic disease; sex or gender; race or ethnicity; socioeconomic indicators explicitly reported by the authors; rurality; language; and digital access or literacy. No population was classified as underserved or vulnerable solely by reviewer inference. Human oversight was charted only when explicitly described and was separated into professional role (eg, nurse, physician or oncologist, psychologist or other mental health professional, multidisciplinary clinical team, researcher or technical team, other expert, or unspecified) and oversight function (content or intervention development, content validation, response review, real-time monitoring, clinical supervision, escalation or referral, or other). Safety governance items included ethics-related procedures, privacy or data security, human or expert oversight, accuracy or hallucination management, crisis escalation or referral, AI identity disclosure, role boundaries, and statements that the system did not replace professional care.

Psychological support functions were operationalized as 7 non–mutually exclusive categories: (1) information or education—provision or explanation of cancer-related, treatment-related, survivorship, psychosocial, or supportive care knowledge; (2) navigation or resource recommendation—direction to services, professionals, pathways, or external resources, including referral options; (3) emotional support—empathic, validating, reassuring, or affect-focused dialogue intended to address emotional experience; (4) motivation or patient activation—goal setting, encouragement, reminders, or other strategies intended to strengthen engagement, confidence, or readiness to act; (5) structured psychological or behavioral intervention—delivery of an identifiable intervention element, such as cognitive behavioral therapy techniques, mindfulness, relaxation, behavioral activation, problem-solving, or another structured therapeutic exercise; (6) disclosure or communication facilitation—elicitation, rehearsal, or support for expressing sensitive concerns or communicating with clinicians or others; and (7) symptom or self-management support—assessment, tracking, feedback, or guidance supporting management of symptoms, treatment effects, health behaviors, or daily functioning.

For theory and therapeutic foundations, 3 mutually exclusive report-level categories were used: (1) an explicit theoretical or conceptual framework, when the report named a framework and described its role in intervention or system development, content, evaluation, or interpretation; (2) therapeutic models or components without an explicit theory-guided framework, when techniques such as cognitive behavioral therapy, mindfulness, motivational interviewing, or relaxation were described, but their use as an overarching development framework was not explicitly stated; and (3) no explicit theoretical basis reported. The presence of a therapeutic technique was not, by itself, coded as a theory-informed intervention development.

Outcome domains were also coded as non–mutually exclusive and included feasibility; usability; acceptability, satisfaction, or user experience; engagement, use, or adherence; psychological or psychosocial outcomes; knowledge, attitudes, empowerment, activation, or self-efficacy; behavioral or self-management outcomes; symptoms, sleep, or quality of life; AI response quality; outcomes related to health care professionals or clinical workflows; and safety or adverse events. To distinguish implemented functions from proposed or hypothetical capabilities, supporting evidence was additionally classified as delivered in an implemented system, planned in a protocol, tested in a simulated patient-facing evaluation, anticipated or prioritized in co-design or formative work, participant-reported use of a nonfixed general-purpose system, or not explicitly described. Coding was based on explicit full-text statements; titles, abstracts, author affiliations, and general background statements were not used to infer unreported features.

Following peer review, the review team refined the charting of support functions, theoretical foundations, system types, human oversight, and population characteristics across the 24 included reports. Uncertain codes were reassessed against the operational definitions, with disagreements and supporting source locations documented. The review questions and eligibility criteria were unchanged.

### Synthesis of Results

We used descriptive numerical summaries and narrative synthesis. Reports were the unit for publication characteristics, study design, theory, outcomes, population characteristics, and governance reporting; underlying projects were the unit for system forms and support functions. Study units identified distinct investigations within or across reports but were not denominators for the principal frequency summaries. Project linkage considered system names and descriptions, authorship, registrations, samples, settings, content, and study periods. Linked reports were counted once per project, with a feature assigned when at least 1 linked report described it. Support, outcome, and governance categories were non–mutually exclusive. Formal critical appraisal and meta-analysis were not undertaken because the review aimed to map the evidence rather than estimate effectiveness.

### Ethical Considerations

This review analyzed published and publicly available literature and did not involve the collection of identifiable human participant data. Institutional ethics review and informed consent were therefore not required.

## Results

### Search Results

A total of 3950 records were identified through database and register searches. After removing 1806 duplicate records (45.7% of those identified), 2144 records remained for title and abstract screening, of which 2067 (96.4%) were excluded. The remaining 77 reports were sought for retrieval, and 1 report could not be retrieved. Consequently, 76 full-text reports were assessed for eligibility. Among these, 52 reports (68.4%) were excluded for the following reasons: not involving the target population (n=6), not involving an AI conversational tool (n=9), not reporting psychological support functions or outcomes (n=8), ineligible evidence type (n=23), insufficient information (n=4), and duplicate publication (n=2). Ultimately, 24 reports (31.6% of those assessed as full texts) were included, representing 25 operational study units nested within 23 underlying AI projects. The PRISMA flow diagram is shown in Figure 1.

No formal critical appraisal was undertaken. Frequencies describe reporting patterns and should not be interpreted as measures of study quality, effectiveness, safety, or clinical readiness.

**Figure 1:**
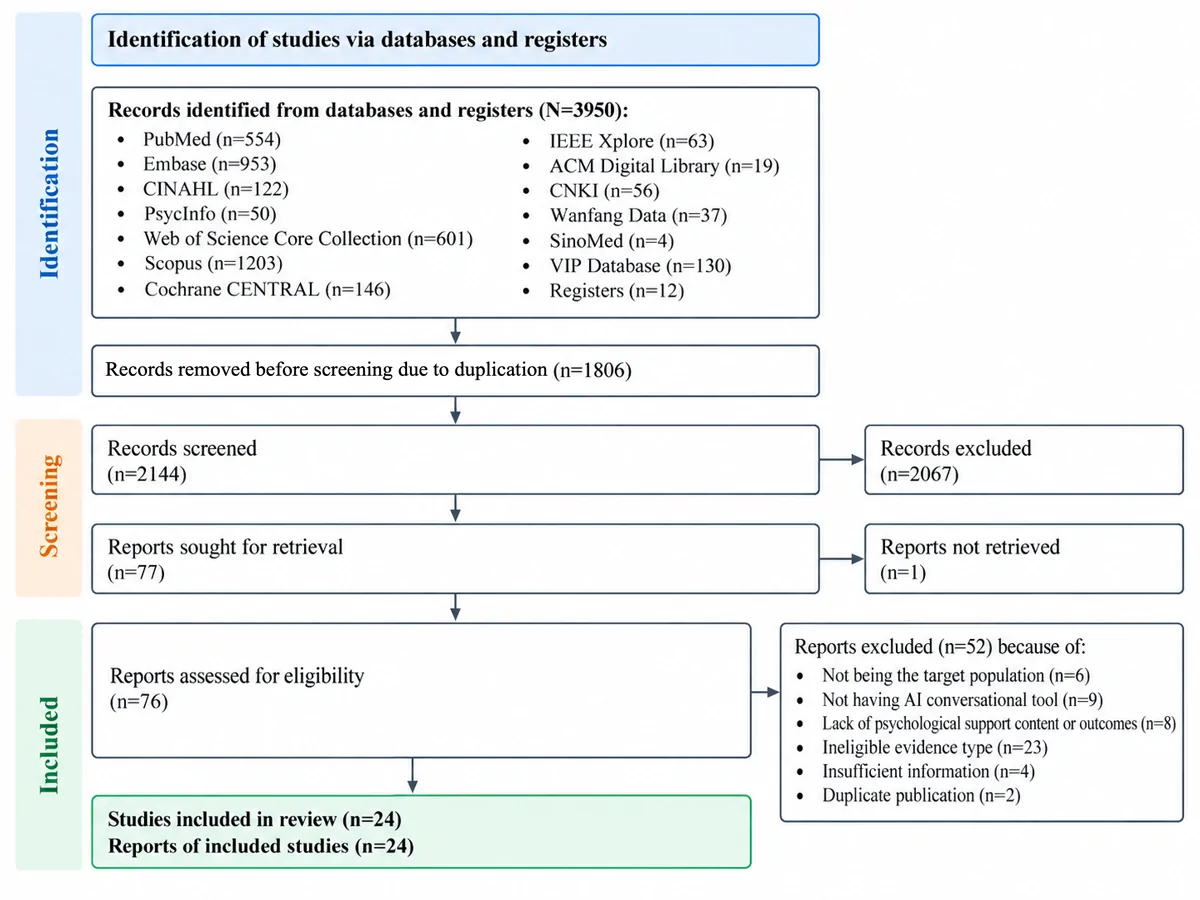
PRISMA (Preferred Reporting Items for Systematic Reviews and Meta-Analyses) flow diagram of evidence selection. The 24 included reports represented 25 operational study units nested within 23 underlying projects.

### Characteristics of Included Reports, Study Units, and Projects

The 24 included reports [11,12,21-42] represented 25 study units and 23 projects (Tables S1 and S2 in Multimedia Appendix 2). The 2 CanRelax reports represented a single study unit and project; the Liu et al [26] and Seo et al [38] reports each contained 2 study units. Of the 24 included reports, 16 (66.7%) were published in 2025 or 2026, and 6 (25%) were from the United States. Designs included 6 randomized or crossover trial reports, 2 trial-derived secondary analyses, 6 nonrandomized user evaluations, and qualitative, co-design, simulated, development, or protocol reports. Table 2 summarizes the main findings; complete descriptive frequencies are provided in Table S3 in Multimedia Appendix 2.

**Table 2:** Selected characteristics and findings of the included reports and projects^a^.

Characteristics			Reports and projects, n (%)
**Primary AI system form (n=23 projects)**			
	Large language model or generative AI	9 (39.1)	
	Rule-, knowledge-, or retrieval-based text agent	6 (26.1)	
	Voice assistant or smart speaker	3 (13)	
	Social robot or embodied agent	1 (4.3)	
	Concept or perception; no fixed deployed system	3 (13)	
	Multimodal monitoring platform and chatbot	1 (4.3)	
**Theory** **and** **therapeutic foundation** **s** **(n=24 reports; categories are mutually exclusive)**			
	No explicit theoretical basis reported	12 (50)	
	Therapeutic models or components without an explicit theory-guided framework	6 (25)	
	Explicit theoretical or conceptual framework	6 (25)	
**Psychological support function** **s** **(any mapped evidence; n=23 projects; categories are non–mutually exclusive)**			
	Information or education	23 (100)	
	Navigation or resource recommendation	14 (60.9)	
	Emotional support	12 (52.2)	
	Motivation or patient activation	10 (43.5)	
	Structured psychological or behavioral intervention	7 (30.4)	
	Disclosure or communication facilitation	6 (26.1)	
	Symptom or self-management support	20 (87)	
**Safety or governance reporting (n=24 reports; explicit reporting only)**			
	Ethics-related procedures	22 (91.7)	
	Human or expert oversight	15 (62.5)	
	Privacy or data protection	13 (54.2)	
	Accuracy or hallucination control	12 (50)	
	Crisis escalation, referral, or high-risk response	8 (33.3)	
	User-facing AI identity disclosure	3 (12.5)	
	Explicit role boundaries	6 (25)	
	Explicit nonreplacement statement	5 (20.8)	

^a^System forms and support functions use 23 projects; theory and governance use 24 reports. Support functions and governance features are non–mutually exclusive. Support function counts include all evidence states; delivered-only frequencies and complete results are presented in Table S3 in Multimedia Appendix 2.

### Characteristics of Conversational AI Agents

The 23 underlying AI projects varied in architecture, delivery mode, interaction modality, automation level, and care integration. LLM or generative AI systems were the most common project-level form (9/23, 39.1%), followed by rule-, knowledge-, or retrieval-based text agents (n=6, 26.1%). Voice assistants or smart speakers accounted for 13% (3/23) of projects, conceptual or nondeployed systems for 13% (n=3), and social robot or embodied systems and multimodal monitoring-plus-chatbot projects for 4.3% (n=1) each. LLM or generative systems included general-purpose models used in oncology support contexts as well as purpose-built or fine-tuned systems. These classifications were based on the reported system or project rather than on model brand alone.

Delivery platforms ranged from web-based generative AI tools and mobile apps to SMS, messaging platforms, smart speakers, social robots, and clinical or monitoring systems with dashboards. Text-based interaction was common, particularly for LLM-based and mobile chatbots, whereas voice interaction was mainly used in smart speaker systems for sleep or symptom support. Some projects integrated multimodal features, passive data collection, or clinical monitoring functions.

Automation levels ranged from fully automated systems to human-supervised or hybrid models. Some projects incorporated clinician oversight, expert validation, or alert-based escalation, whereas other reports described stand-alone, simulated, conceptual, or participant-experienced systems. Care integration also varied, with agents functioning as stand-alone tools or being embedded in mobile health (mHealth) interventions, survivorship care, or clinical workflows. The technical and implementation characteristics of the projects are summarized in Multimedia Appendix 2.

### Psychological Support Functions

Psychological support functions were diverse and non–mutually exclusive. Across the 23 projects, information or education was mapped in 23 (100%), symptom or self-management support in 20 (87%), navigation or resource recommendation in 14 (60.9%), emotional support in 12 (52.2%), motivation or patient activation in 10 (43.5%), structured psychological or behavioral intervention in 7 (30.4%), and disclosure or communication facilitation in 6 (26.1%). These frequencies include delivered, planned, simulated, anticipated or co-designed, and participant-reported evidence states. In a delivered-only sensitivity analysis, the corresponding frequencies were 13 (56.5%), 12 (52.2%), 6 (26.1%), 5 (21.7%), 7 (30.4%), 5 (21.7%), and 1 (4.3%), respectively. Figure 2 shows support functions by primary system form.

**Figure 2:**
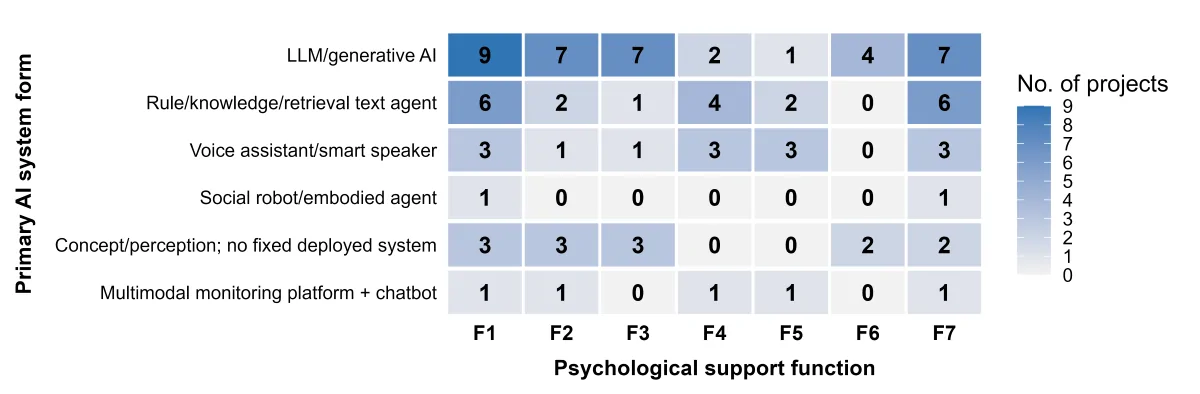
Heatmap summarizing project-level data from 23 AI projects. Cells show project counts, and functions are not mutually exclusive. Evidence states included delivered, planned, simulated, anticipated/co-designed, and participant-reported functions. Each project contributed once to each applicable cell. LLM: large language model.

Emotional support concerned empathic or validating dialogue; motivation or patient activation concerned engagement or readiness to act; structured psychological or behavioral intervention required an identifiable therapeutic technique or exercise. These functions could co-occur. Information or education and navigation or resource recommendation were distinguished as knowledge provision and direction to services or resources, respectively.

Quality of life was treated as an outcome or goal, and sensitive topics as contextual tags. Support functions were not inferred from outcome measurement alone.

### Outcomes and Evaluation Approaches

Across the 24 reports, acceptability, satisfaction, or user experience was the most frequently reported evaluation domain (n=17, 70.8%), followed by engagement, use, or adherence (n=13, 54.2%). Feasibility was reported in 6 (25%) reports and usability in 8 (33.3%).

Psychological or psychosocial clinical outcomes were reported or planned in 8 (33.3%) reports. Knowledge, attitudes, empowerment, patient activation, or self-efficacy outcomes were reported in 8 (33.3%), behavioral or self-management outcomes in 9 (37.5%), and symptom, sleep, or quality-of-life outcomes in 6 (25%). These domains included standardized and study-specific measures.

AI response quality, factuality, empathy, readability, inclusiveness, bias, hallucination, or contextual error outcomes were reported in 29.2% (7/24) of reports. Outcomes related to health care professionals or clinical workflows were reported in 5 (20.8%) reports, and safety or adverse event evaluation in 4 (16.7%).

### Theory and Therapeutic Foundations

An explicit theoretical or conceptual framework was reported in 25% (6/24) of reports; 6 (25%) reports described therapeutic components without an explicit theory-guided framework; and 12 (50%) reports reported neither. Named frameworks supported development, co-design, evaluation, or interpretation. Therapeutic components included cognitive behavioral therapy, mindfulness, relaxation, motivational interviewing, sleep hygiene, self-monitoring, and feedback. Framework reporting did not necessarily indicate theory-guided intervention development.

### Safety, Ethics, and Governance

Of the 24 included reports, ethics-related procedures were reported in 22 (91.7%), human or expert oversight in 15 (62.5%), privacy or data protection in 13 (54.2%), and accuracy or hallucination management in 12 (50%). Figure 3 presents governance reporting by primary system form at the project level.

Crisis escalation, referral, or high-risk response was described in 33.3% (8/24) of reports, user-facing AI identity disclosure in 12.5% (n=3), role boundaries in 25% (n=6), and nonreplacement statements in 20.8% (n=5). Table S2 in Multimedia Appendix 2 separates professional roles from their functions and distinguishes system oversight from other research activities.

**Figure 3:**
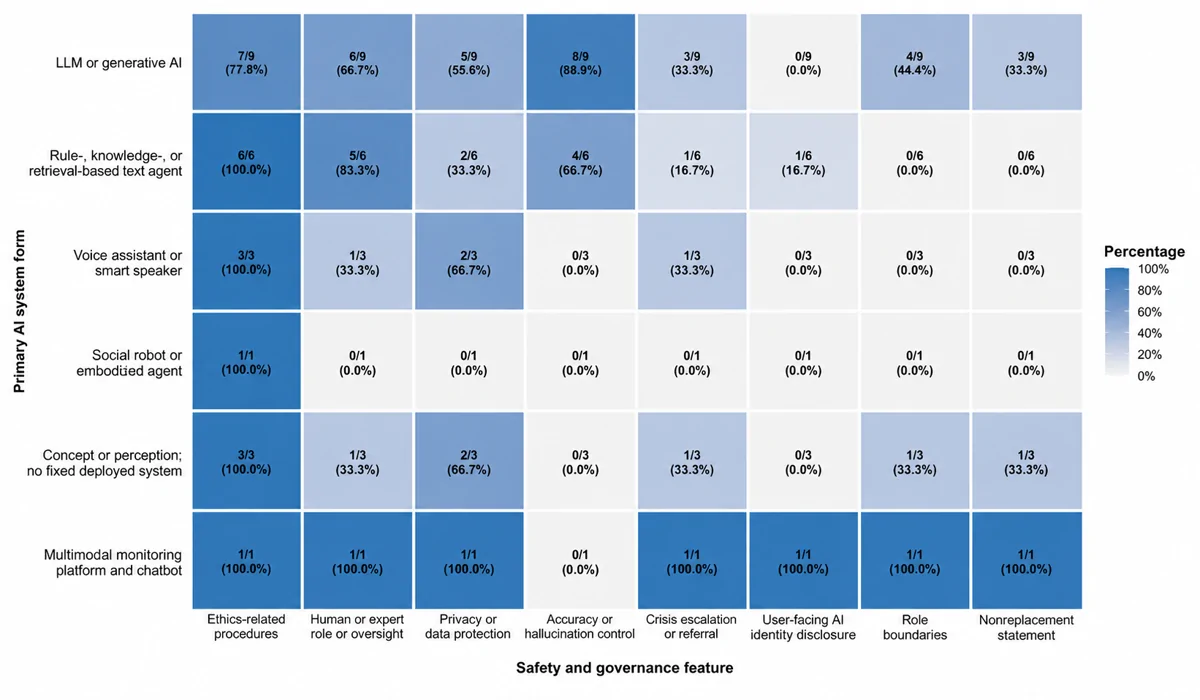
Safety and governance features reported by primary AI system form. Cells show the number and percentage of projects as n/N (%) within each system form category across 23 projects. Each project was counted once when at least 1 linked report described the feature; linked reports from the same project were not counted separately. Categories were non–mutually exclusive, and features not explicitly reported were not assumed to be absent. Report-level frequencies are provided in Table 2. LLM: large language model.

### Population Representation and Equity

Population and equity-related characteristics were inconsistently reported across the 24 reports. Pediatric or adolescent and young adult representation was explicit in 6 (25%) reports, adults aged ≥65 years in 7 (29.2%), and advanced or metastatic disease in 4 (16.7%). Race or ethnicity was reported in 4 (16.7%) reports; education, occupation, or income-related variables in 14 (58.3%); explicit language eligibility or context in 16 (66.7%); and digital access or literacy in 11 (45.8%). No report established participant-level rurality (n=0).

## Discussion

### Principal Findings and Contribution

This review identified 24 reports on conversational AI for psychological support in cancer care. Information or education and symptom or self-management functions predominated, whereas delivered structured psychological interventions were less common. Evaluation emphasized acceptability and engagement, with psychosocial clinical and safety assessments reported less frequently. Theoretical foundations, population characteristics, and operational oversight were incompletely reported.

The review complements broader digital mental health and oncology AI syntheses [5,9,13,14,18] by linking conversational system forms to explicitly defined support functions and distinguishing delivered from proposed or simulated capabilities. Its inclusion of nontrial evidence also captures development, user perspectives, and governance questions that a randomized controlled trial (RCT)–focused synthesis cannot fully address.

### Technologies and Psychological Support Functions

The coexistence of LLM or generative AI systems with rule-, knowledge-, or retrieval-based agents, voice assistants, embodied systems, and multimodal platforms indicates that conversational AI in psycho-oncology should not be treated as a single intervention class. This is consistent with the cancer care–wide review by Jiang et al [9] and the broader health care chatbot literature [13], which describe substantial heterogeneity in delivery modes, interaction logic, and implementation characteristics. A recent systematic review of 160 mental health chatbot studies likewise documented a rapid transition from rule-based systems toward LLM-based approaches, while noting that most LLM studies remained at early validation stages [43]. These findings matter because system architecture affects the balance between conversational flexibility and content control: more open-ended generation may support personalization and empathic interaction, whereas more constrained systems may permit tighter control over content and response pathways. Comparisons across systems therefore need to distinguish technological form from clinical maturity or effectiveness.

Information and self-management functions may be easier to anchor to curated content or predefined workflows than sustained psychological interventions, which require therapeutic continuity and responses to deterioration or high-risk disclosures. This may help explain their wider representation, consistent with broader oncology evidence [9]. The smaller delivered-only counts also show why proposed and simulated capabilities should be distinguished from implemented functions.

### Evaluation Outcomes and Evidence Maturity

Acceptability, user experience, and engagement were reported more often than psychosocial clinical or safety outcomes, consistent with broader oncology and mental health chatbot evidence [9,43]. These non–mutually exclusive domains often co-occurred; the comparison concerns reporting frequency rather than separate types of study.

The pattern is also consistent with the developmental stage of the field. Many included reports were feasibility or pilot studies, co-design studies, simulated response evaluations, system development studies, or protocols, for which usability, acceptability, and engagement are appropriate early-stage outcomes. Broader mental health evidence suggests that efficacy is beginning to emerge but remains uncertain. A 2025 systematic review and meta-analysis of generative AI mental health chatbots found a modest average reduction in negative mental health outcomes but with substantial heterogeneity and wide prediction intervals [44]. A subsequent 2026 systematic review of general-purpose LLMs in mental health care found that most evidence came from vignette, simulation, or retrospective studies, with sparse prospective real-world evaluation and generally low certainty across clinical domains [45]. These findings support a distinction between demonstrating that a system can be used or is acceptable and demonstrating durable psychosocial benefit, safety, and clinical utility. Future evaluations should therefore progress from formative testing to prospective studies that align outcomes with the intended support function, distinguish process measures from patient outcomes, and assess workflow and safety consequences when clinical integration is intended.

### Theory and Psychosocial Mechanisms

Only one-quarter of reports named a theoretical or conceptual framework. This provides a complementary perspective to outcome-focused reviews [5,18,44], but neither a therapeutic component nor a named framework demonstrates that a psychosocial mechanism was tested.

The varied disciplinary origins and developmental purposes of these systems may contribute to inconsistent theory reporting. Future studies should link support functions to proposed mechanisms, such as self-efficacy, emotion regulation, reduced avoidance, or help-seeking, and prespecify corresponding outcomes. This would clarify how conversational components are intended to work.

### Population Representation and Equity

Population and equity-related reporting was insufficient to determine how representative the evidence base is. Older adults, people with advanced or metastatic disease, and several characteristics relevant to equity, including race or ethnicity, rurality, language, and digital access or literacy, were incompletely reported. The central concern is therefore not the demonstrated exclusion of these groups but the limited ability to judge who can access, use, and benefit from the systems studied. Broader generative AI mental health evidence has similarly identified limited study of older adults [44]. In oncology specifically, a 2026 qualitative systematic review found that older adults with cancer can value digital interventions for information access and self-management but also encounter technical difficulties, suboptimal content design, and heterogeneous support needs [46]. A subsequent scoping review of equity in digital oncology reported geographic and population gaps, persistent technical access barriers, and associations between socioeconomic factors and uptake [47].

Formative and simulated studies may prioritize interaction quality over representativeness. Nevertheless, age, functional burden, language, digital access, and support needs can affect use in oncology [46,47]. Studies should report relevant participant characteristics and eligibility requirements, justify exclusions, and plan subgroup analyses when feasible.

### Safety Governance and Human Oversight

Safety and governance reporting showed a notable imbalance: conventional ethics-related procedures and some form of human or expert involvement were reported more often than operational safeguards such as crisis escalation, AI identity disclosure, explicit role boundaries, and nonreplacement statements. One possible explanation is that institutional research ethics and privacy procedures are already embedded in established research governance, whereas AI-specific safeguards for open-ended conversational systems are newer and less standardized. This interpretation is consistent with a 2026 scoping review of safety mechanisms in generative AI mental health chatbots, which found that crisis referral protocols were often underdeveloped, systematic adverse event monitoring was sparse, and human oversight during intervention delivery was limited [48]. General-purpose LLM evaluations have also documented unsafe responses to patient-posed medical questions [17], reinforcing the need to treat safety as an empirical outcome rather than an assumed property of the system.

Human oversight encompassed development, content validation, response review, monitoring, supervision, and referral. Thus, 15 of 24 reports describing some oversight does not imply 15 clinically monitored systems. Evidence on LLMs in mental health similarly warrants caution about unsupervised high-risk use [45]. Cancer distress guidance supports clear assessment and referral pathways [49,50], while DECIDE-AI (Developmental and Exploratory Clinical Investigations of Decision Support Systems Driven by Artificial Intelligence), SPIRIT (Standard Protocol Items: Recommendations for Interventional Trials)-AI, and CONSORT (Consolidated Standards of Reporting Trials)-AI provide reporting frameworks for responsibilities, monitoring, and safety evaluation [51-53].

### Implications for Psycho-Oncology Practice and Care Delivery

Taken together, the findings support a risk- and function-sensitive role for conversational AI within multidisciplinary psycho-oncology and supportive care pathways. Information, education, navigation, and selected self-management functions may complement existing care by extending access to standardized support and reinforcing patient preparation between clinical encounters. More complex emotional support or structured psychological intervention requires stronger evidence of therapeutic benefit, continuity, and safety, particularly when systems may encounter severe distress or other high-risk disclosures. The current evidence therefore supports evaluating conversational AI as an adjunct to, rather than a substitute for, professional psycho-oncology care.

Professional responsibilities should match system functions: mental health professionals may guide therapeutic content and risk assessment; oncology clinicians may guide disease-specific accuracy; nurses may guide education, symptom monitoring, counseling, and coordination; and technical teams may guide validation and incident management. Included reports illustrate nursing contributions to development, co-design, validation, or monitoring [26,29,40,42], without establishing the effectiveness of nurse-AI collaboration. Implementation studies should specify responsibility for human review, referral, documentation, and incident response.

### Strengths and Limitations

Strengths include a registered protocol, multidisciplinary search, independent screening and charting, and explicit linkage of reports, study units, and projects. Separating delivered functions from other evidence states improves the interpretability of the evidence map.

The search ended on May 5, 2026, and may not capture later publications, commercial systems, or nonindexed work. Later reviews were used only for context. Included designs ranged from protocols and simulated evaluations to trials, so mapped projects do not represent equivalent levels of clinical evidence. Without formal critical appraisal or meta-analysis, the review cannot establish effectiveness, safety, or certainty of evidence.

Charting relied on explicit reporting and may underestimate undocumented theoretical, technical, population, or governance features. Incomplete project identifiers may also affect report linkage. General-purpose LLMs were eligible only within studied oncology support contexts; findings therefore cannot characterize unstudied, independent everyday use by people with cancer.

### Conclusions

Conversational AI in cancer psychological support encompasses diverse technologies and functions, with more evidence on information provision and self-management than on delivered structured psychological interventions. Future evaluations should test psychosocial benefit and safety, clarify theoretical mechanisms, report population characteristics, and define professional oversight and referral responsibilities. The present evidence map does not establish clinical effectiveness or readiness for routine use.

## Data Availability

The review data were derived from the cited reports. Search strategies are provided in Multimedia Appendix 1. Report and project characteristics, evidence-unit linkage, and descriptive frequencies are provided in Multimedia Appendix 2. Source-located charting records are available from the corresponding author upon reasonable request.

## Appendix

Multimedia Appendix 1 Search strategies.

Multimedia Appendix 2 Characteristics and final functional coding of the included reports; project-level system, function, theory, and human-oversight mapping, and complete descriptive frequencies.

Multimedia Appendix 3 PRISMA-ScR checklist.

## Abbreviations

CONSORT: Consolidated Standards of Reporting Trials
DECIDE-AI: Developmental and Exploratory Clinical Investigations of Decision Support Systems Driven by Artificial Intelligence
JBI: Joanna Briggs Institute
LLM: large language model
mHealth: mobile health
PRISMA: Preferred Reporting Items for Systematic Reviews and Meta-Analyses
PRISMA-ScR: Preferred Reporting Items for Systematic Reviews and Meta-Analyses extension for Scoping Reviews
RCT: randomized controlled trial
SPIRIT: Standard Protocol Items: Recommendations for Interventional Trials
WHO: World Health Organization
