# Supplementary material for: Mapping Technologies, Support Functions, Evaluation Outcomes, and Safety Governance Related to Conversational AI Agents for Psychological Support of Patients With Cancer: Scoping Review
Source: J Med Internet Res. 2026 Oct 19;28:e105599. doi: 10.2196/105599 (PMC13728763; doi:10.2196/105599)
Supplement: Supplementary file 1 [file jmir_v28i1e105599_app1.docx]

**Search Strategies**

**PubMed:2026/05/06**

| **Search set** | **Search terms** |
| --- | --- |
| **#1 AI-based conversational agents**  **(38291)** | chatbot*[tiab] OR "chat bot*"[tiab] OR chatterbot*[tiab] OR "AI bot*"[tiab] OR "chat robot*"[tiab] OR "conversational agent*"[tiab] OR "conversational AI"[tiab] OR "conversational system*"[tiab] OR "dialogue system*"[tiab] OR "dialog system*"[tiab] OR "virtual agent*"[tiab] OR "digital agent*"[tiab] OR "digital assistant*"[tiab] OR "virtual assistant*"[tiab] OR "intelligent assistant*"[tiab] OR "voice assistant*"[tiab] OR "relational agent*"[tiab] OR "embodied conversational agent*"[tiab] OR "social robot*"[tiab] OR "social bot*"[tiab] OR "AI companion*"[tiab] OR "virtual coach*"[tiab] OR "digital coach*"[tiab] OR "e-coach*"[tiab] OR "nurse avatar*"[tiab] OR "nursing avatar*"[tiab] OR "virtual avatar*"[tiab] OR "conversational avatar*"[tiab] OR "large language model*"[tiab] OR "language model*"[tiab] OR "foundation model*"[tiab] OR LLM[tiab] OR LLMs[tiab] OR "generative AI"[tiab] OR "generative artificial intelligence"[tiab] OR "generative pre-trained transformer*"[tiab] OR "generative pretrained transformer*"[tiab] OR "AI agent*"[tiab] OR "artificial intelligence agent*"[tiab] OR "agentic AI"[tiab] OR ChatGPT[tiab] OR "GPT-3*"[tiab] OR "GPT-4*"[tiab] OR "GPT-5*"[tiab] OR OpenAI[tiab] OR "Bing Chat"[tiab] OR "Google Bard"[tiab] OR "Bard AI"[tiab] OR "Google Gemini"[tiab] OR "Gemini AI"[tiab] OR Claude[tiab] OR "Claude AI"[tiab] OR "Microsoft Copilot"[tiab] OR "question answering"[tiab] OR "question-answering"[tiab] OR "question answer* system*"[tiab] OR Vivibot[tiab] OR (( "Artificial Intelligence"[Mesh] OR "Generative Artificial Intelligence"[Mesh] OR "Natural Language Processing"[Mesh] OR "Robotics"[Mesh] ) AND ( chatbot*[tiab] OR "chat bot*"[tiab] OR chatterbot*[tiab] OR "AI bot*"[tiab] OR "chat robot*"[tiab] OR "conversational agent*"[tiab] OR "conversational AI"[tiab] OR "conversational system*"[tiab] OR "dialogue system*"[tiab] OR "dialog system*"[tiab] OR "virtual agent*"[tiab] OR "digital assistant*"[tiab] OR "virtual assistant*"[tiab] OR "intelligent assistant*"[tiab] OR "voice assistant*"[tiab] OR "relational agent*"[tiab] OR "embodied conversational agent*"[tiab] OR "social robot*"[tiab] OR "AI companion*"[tiab] OR "large language model*"[tiab] OR "language model*"[tiab] OR "foundation model*"[tiab] OR LLM[tiab] OR LLMs[tiab] OR "generative AI"[tiab] OR "AI agent*"[tiab] OR OpenAI[tiab] OR ChatGPT[tiab] )) |
| **#2 Cancer**  **(5920690)** | "Neoplasms"[Mesh] OR "Cancer Survivors"[Mesh] OR cancer*[tiab] OR neoplasm*[tiab] OR neoplastic[tiab] OR neoplasia*[tiab] OR tumor*[tiab] OR tumour*[tiab] OR carcinoma*[tiab] OR malignan*[tiab] OR oncolog*[tiab] OR leukemia*[tiab] OR leukaemia*[tiab] OR lymphoma*[tiab] OR melanoma*[tiab] OR sarcoma*[tiab] OR blastoma*[tiab] OR myeloma*[tiab] OR "solid tumor*"[tiab] OR "solid tumour*"[tiab] OR "hematologic malignan*"[tiab] OR "haematologic malignan*"[tiab] OR "cancer survivor*"[tiab] OR "cancer survivorship"[tiab] |
| **#3 Psychological support-related terms**  **(4112585)** | "Psychological Distress"[Mesh] OR "Psycho-Oncology"[Mesh] OR "Psychosocial Support Systems"[Mesh] OR "Mental Health"[Mesh] OR "Psychotherapy"[Mesh] OR "Cognitive Behavioral Therapy"[Mesh] OR "Behavior Therapy"[Mesh] OR "Counseling"[Mesh] OR "Mindfulness"[Mesh] OR "Social Support"[Mesh] OR "Stress, Psychological"[Mesh] OR "Adaptation, Psychological"[Mesh] OR "Anxiety"[Mesh] OR "Depression"[Mesh] OR "Depressive Disorder"[Mesh] OR "Quality of Life"[Mesh] OR "Palliative Care"[Mesh] OR psycholog*[tiab] OR psychosocial[tiab] OR psycho-oncolog*[tiab] OR psychooncolog*[tiab] OR "mental health"[tiab] OR "mental wellbeing"[tiab] OR "mental well-being"[tiab] OR "psychological distress"[tiab] OR "emotional distress"[tiab] OR distress[tiab] OR anxiety[tiab] OR anxious[tiab] OR depression[tiab] OR depressive[tiab] OR stress[tiab] OR loneliness[tiab] OR "emotional wellbeing"[tiab] OR "emotional well-being"[tiab] OR wellbeing[tiab] OR well-being[tiab] OR "quality of life"[tiab] OR "psychological support"[tiab] OR "psychosocial support"[tiab] OR "emotional support"[tiab] OR "mental health support"[tiab] OR "psychological care"[tiab] OR "psychosocial care"[tiab] OR "emotional care"[tiab] OR "psychosocial oncology"[tiab] OR "psycho-oncology"[tiab] OR empath*[tiab] OR reassurance[tiab] OR "therapeutic communication"[tiab] OR "supportive communication"[tiab] OR counseling[tiab] OR counselling[tiab] OR psychotherapy[tiab] OR psychoeducation[tiab] OR "psychological intervention*"[tiab] OR "psychosocial intervention*"[tiab] OR "behavioral intervention*"[tiab] OR "behavioural intervention*"[tiab] OR "cognitive behavioral therap*"[tiab] OR "cognitive behavioural therap*"[tiab] OR CBT[tiab] OR mindfulness[tiab] OR relaxation[tiab] OR "problem solving"[tiab] OR "positive psychology"[tiab] OR "positive affect"[tiab] OR resilience[tiab] OR coping[tiab] OR adjustment[tiab] OR "psychological adjustment"[tiab] OR "fear of cancer recurrence"[tiab] OR "fear of recurrence"[tiab] OR "fear of progression"[tiab] OR "cancer worry"[tiab] OR "illness uncertainty"[tiab] OR "uncertainty management"[tiab] OR "cancer-related uncertainty"[tiab] OR "body image"[tiab] OR "fertility concern*"[tiab] OR "sexual health"[tiab] OR intimacy[tiab] OR "unmet need*"[tiab] OR "supportive care need*"[tiab] OR "supportive care"[tiab] OR "treatment engagement"[tiab] OR "patient activation"[tiab] OR "self-management"[tiab] OR "self management"[tiab] OR "self-efficacy"[tiab] OR "self efficacy"[tiab] OR "patient education"[tiab] OR "health education"[tiab] OR "informational support"[tiab] OR "information support"[tiab] OR "survivorship care"[tiab] OR "palliative care"[tiab] |
| **#4 Primary focused search**  **(554)** | (#1 AND #2 AND #3) NOT (animals[mh] NOT humans[mh]) |
| **#5 Sensitivity search**  **(3261)** | (#1 AND #2) NOT (animals[mh] NOT humans[mh]) |

**Embase:2026/05/06**

| Search set | Search terms |
| --- | --- |
| **#1 AI-based conversational agents**  **(31994)** | chatbot*:ti,ab,kw OR 'chat bot*':ti,ab,kw OR chatterbot*:ti,ab,kw OR 'ai bot*':ti,ab,kw OR 'chat robot*':ti,ab,kw OR 'conversational agent*':ti,ab,kw OR 'conversational ai':ti,ab,kw OR 'conversational system*':ti,ab,kw OR 'dialogue system*':ti,ab,kw OR 'dialog system*':ti,ab,kw OR 'virtual agent*':ti,ab,kw OR 'digital agent*':ti,ab,kw OR 'digital assistant*':ti,ab,kw OR 'virtual assistant*':ti,ab,kw OR 'intelligent assistant*':ti,ab,kw OR 'voice assistant*':ti,ab,kw OR 'relational agent*':ti,ab,kw OR 'embodied conversational agent*':ti,ab,kw OR 'social robot*':ti,ab,kw OR 'social bot*':ti,ab,kw OR 'ai companion*':ti,ab,kw OR 'virtual coach*':ti,ab,kw OR 'digital coach*':ti,ab,kw OR 'e-coach*':ti,ab,kw OR 'nurse avatar*':ti,ab,kw OR 'nursing avatar*':ti,ab,kw OR 'virtual avatar*':ti,ab,kw OR 'conversational avatar*':ti,ab,kw OR 'large language model*':ti,ab,kw OR 'language model*':ti,ab,kw OR 'foundation model*':ti,ab,kw OR llm:ti,ab,kw OR llms:ti,ab,kw OR 'generative ai':ti,ab,kw OR 'generative artificial intelligence':ti,ab,kw OR 'generative pre-trained transformer*':ti,ab,kw OR 'generative pretrained transformer*':ti,ab,kw OR 'ai agent*':ti,ab,kw OR 'artificial intelligence agent*':ti,ab,kw OR 'agentic ai':ti,ab,kw OR chatgpt:ti,ab,kw OR 'gpt-3*':ti,ab,kw OR 'gpt-4*':ti,ab,kw OR 'gpt-5*':ti,ab,kw OR openai:ti,ab,kw OR 'bing chat':ti,ab,kw OR 'google bard':ti,ab,kw OR 'bard ai':ti,ab,kw OR 'google gemini':ti,ab,kw OR 'gemini ai':ti,ab,kw OR claude:ti,ab,kw OR 'claude ai':ti,ab,kw OR 'microsoft copilot':ti,ab,kw OR 'question answering':ti,ab,kw OR 'question-answering':ti,ab,kw OR 'question answer* system*':ti,ab,kw OR vivibot:ti,ab,kw |
| **#2 Cancer**  **(8592560)** | 'neoplasm'/exp OR 'cancer survivor'/exp OR cancer*:ti,ab,kw OR neoplasm*:ti,ab,kw OR neoplastic:ti,ab,kw OR neoplasia*:ti,ab,kw OR tumor*:ti,ab,kw OR tumour*:ti,ab,kw OR carcinoma*:ti,ab,kw OR malignan*:ti,ab,kw OR oncolog*:ti,ab,kw OR leukemia*:ti,ab,kw OR leukaemia*:ti,ab,kw OR lymphoma*:ti,ab,kw OR melanoma*:ti,ab,kw OR sarcoma*:ti,ab,kw OR blastoma*:ti,ab,kw OR myeloma*:ti,ab,kw OR 'solid tumor*':ti,ab,kw OR 'solid tumour*':ti,ab,kw OR 'hematologic malignan*':ti,ab,kw OR 'haematologic malignan*':ti,ab,kw OR 'cancer survivor*':ti,ab,kw OR 'cancer survivorship':ti,ab,kw |
| **#3 Psychological support-related terms**  **(6119712)** | 'psychological distress'/exp OR 'psychooncology'/exp OR 'mental health'/exp OR 'psychotherapy'/exp OR 'cognitive therapy'/exp OR 'behavior therapy'/exp OR 'counseling'/exp OR 'mindfulness'/exp OR 'social support'/exp OR 'psychological stress'/exp OR 'coping behavior'/exp OR 'adaptation'/exp OR 'anxiety'/exp OR 'depression'/exp OR 'depressive disorder'/exp OR 'quality of life'/exp OR 'palliative therapy'/exp OR psycholog*:ti,ab,kw OR psychosocial:ti,ab,kw OR 'psycho-oncolog*':ti,ab,kw OR psychooncolog*:ti,ab,kw OR 'mental health':ti,ab,kw OR 'mental wellbeing':ti,ab,kw OR 'mental well-being':ti,ab,kw OR 'psychological distress':ti,ab,kw OR 'emotional distress':ti,ab,kw OR distress:ti,ab,kw OR anxiety:ti,ab,kw OR anxious:ti,ab,kw OR depression:ti,ab,kw OR depressive:ti,ab,kw OR stress:ti,ab,kw OR loneliness:ti,ab,kw OR 'emotional wellbeing':ti,ab,kw OR 'emotional well-being':ti,ab,kw OR wellbeing:ti,ab,kw OR 'well-being':ti,ab,kw OR 'quality of life':ti,ab,kw OR 'psychological support':ti,ab,kw OR 'psychosocial support':ti,ab,kw OR 'emotional support':ti,ab,kw OR 'mental health support':ti,ab,kw OR 'psychological care':ti,ab,kw OR 'psychosocial care':ti,ab,kw OR 'emotional care':ti,ab,kw OR 'psychosocial oncology':ti,ab,kw OR 'psycho-oncology':ti,ab,kw OR empath*:ti,ab,kw OR reassurance:ti,ab,kw OR 'therapeutic communication':ti,ab,kw OR 'supportive communication':ti,ab,kw OR counseling:ti,ab,kw OR counselling:ti,ab,kw OR psychotherapy:ti,ab,kw OR psychoeducation:ti,ab,kw OR 'psychological intervention*':ti,ab,kw OR 'psychosocial intervention*':ti,ab,kw OR 'behavioral intervention*':ti,ab,kw OR 'behavioural intervention*':ti,ab,kw OR 'cognitive behavioral therap*':ti,ab,kw OR 'cognitive behavioural therap*':ti,ab,kw OR cbt:ti,ab,kw OR mindfulness:ti,ab,kw OR relaxation:ti,ab,kw OR 'problem solving':ti,ab,kw OR 'positive psychology':ti,ab,kw OR 'positive affect':ti,ab,kw OR resilience:ti,ab,kw OR coping:ti,ab,kw OR adjustment:ti,ab,kw OR 'psychological adjustment':ti,ab,kw OR 'fear of cancer recurrence':ti,ab,kw OR 'fear of recurrence':ti,ab,kw OR 'fear of progression':ti,ab,kw OR 'cancer worry':ti,ab,kw OR 'illness uncertainty':ti,ab,kw OR 'uncertainty management':ti,ab,kw OR 'cancer-related uncertainty':ti,ab,kw OR 'body image':ti,ab,kw OR 'fertility concern*':ti,ab,kw OR 'sexual health':ti,ab,kw OR intimacy:ti,ab,kw OR 'unmet need*':ti,ab,kw OR 'supportive care need*':ti,ab,kw OR 'supportive care':ti,ab,kw OR 'treatment engagement':ti,ab,kw OR 'patient activation':ti,ab,kw OR 'self-management':ti,ab,kw OR 'self management':ti,ab,kw OR 'self-efficacy':ti,ab,kw OR 'self efficacy':ti,ab,kw OR 'patient education':ti,ab,kw OR 'health education':ti,ab,kw OR 'informational support':ti,ab,kw OR 'information support':ti,ab,kw OR 'survivorship care':ti,ab,kw OR 'palliative care':ti,ab,kw |
| **#4 Primary focused search**  **(953)** | (#1 AND #2 AND #3) NOT ('animal'/exp NOT 'human'/exp) |

CINAHL via EBSCOhost:2026/05/06

| Search set | Search terms |
| --- | --- |
| **S1 AI-based conversational agents**  **(9,697)** | TI (chatbot* OR "chat bot*" OR chatterbot* OR "AI bot*" OR "chat robot*" OR "conversational agent*" OR "conversational AI" OR "conversational system*" OR "dialogue system*" OR "dialog system*" OR "virtual agent*" OR "digital agent*" OR "digital assistant*" OR "virtual assistant*" OR "intelligent assistant*" OR "voice assistant*" OR "relational agent*" OR "embodied conversational agent*" OR "social robot*" OR "social bot*" OR "AI companion*" OR "virtual coach*" OR "digital coach*" OR "e-coach*" OR "nurse avatar*" OR "nursing avatar*" OR "virtual avatar*" OR "conversational avatar*" OR "large language model*" OR "language model*" OR "foundation model*" OR LLM OR LLMs OR "generative AI" OR "generative artificial intelligence" OR "generative pre-trained transformer*" OR "generative pretrained transformer*" OR "AI agent*" OR "artificial intelligence agent*" OR "agentic AI" OR ChatGPT OR "GPT-3*" OR "GPT-4*" OR "GPT-5*" OR OpenAI OR "Bing Chat" OR "Google Bard" OR "Bard AI" OR "Google Gemini" OR "Gemini AI" OR Claude OR "Claude AI" OR "Microsoft Copilot" OR "question answering" OR "question-answering" OR "question answer* system*" OR Vivibot)  OR AB (chatbot* OR "chat bot*" OR chatterbot* OR "AI bot*" OR "chat robot*" OR "conversational agent*" OR "conversational AI" OR "conversational system*" OR "dialogue system*" OR "dialog system*" OR "virtual agent*" OR "digital agent*" OR "digital assistant*" OR "virtual assistant*" OR "intelligent assistant*" OR "voice assistant*" OR "relational agent*" OR "embodied conversational agent*" OR "social robot*" OR "social bot*" OR "AI companion*" OR "virtual coach*" OR "digital coach*" OR "e-coach*" OR "nurse avatar*" OR "nursing avatar*" OR "virtual avatar*" OR "conversational avatar*" OR "large language model*" OR "language model*" OR "foundation model*" OR LLM OR LLMs OR "generative AI" OR "generative artificial intelligence" OR "generative pre-trained transformer*" OR "generative pretrained transformer*" OR "AI agent*" OR "artificial intelligence agent*" OR "agentic AI" OR ChatGPT OR "GPT-3*" OR "GPT-4*" OR "GPT-5*" OR OpenAI OR "Bing Chat" OR "Google Bard" OR "Bard AI" OR "Google Gemini" OR "Gemini AI" OR Claude OR "Claude AI" OR "Microsoft Copilot" OR "question answering" OR "question-answering" OR "question answer* system*" OR Vivibot) |
| **S2 Cancer**  **(1,029,449)** | (MH "Neoplasms+")  OR (MH "Cancer Survivors")  OR TI (cancer* OR neoplasm* OR neoplastic OR neoplasia* OR tumor* OR tumour* OR carcinoma* OR malignan* OR oncolog* OR leukemia* OR leukaemia* OR lymphoma* OR melanoma* OR sarcoma* OR blastoma* OR myeloma* OR "solid tumor*" OR "solid tumour*" OR "hematologic malignan*" OR "haematologic malignan*" OR "cancer survivor*" OR "cancer survivorship")  OR AB (cancer* OR neoplasm* OR neoplastic OR neoplasia* OR tumor* OR tumour* OR carcinoma* OR malignan* OR oncolog* OR leukemia* OR leukaemia* OR lymphoma* OR melanoma* OR sarcoma* OR blastoma* OR myeloma* OR "solid tumor*" OR "solid tumour*" OR "hematologic malignan*" OR "haematologic malignan*" OR "cancer survivor*" OR "cancer survivorship") |
| **S3 Psychological support-related terms**  **(1,430,472)** | (MH "Psychological Distress")  OR (MH "Psychosocial Factors")  OR (MH "Mental Health")  OR (MH "Psychotherapy+")  OR (MH "Cognitive Therapy")  OR (MH "Behavior Therapy")  OR (MH "Counseling+")  OR (MH "Mindfulness")  OR (MH "Social Support")  OR (MH "Stress, Psychological")  OR (MH "Adaptation, Psychological")  OR (MH "Anxiety+")  OR (MH "Depression+")  OR (MH "Quality of Life")  OR (MH "Palliative Care")  OR TI (psycholog* OR psychosocial OR psycho-oncolog* OR psychooncolog* OR "mental health" OR "mental wellbeing" OR "mental well-being" OR "psychological distress" OR "emotional distress" OR distress OR anxiety OR anxious OR depression OR depressive OR stress OR loneliness OR "emotional wellbeing" OR "emotional well-being" OR wellbeing OR well-being OR "quality of life" OR "psychological support" OR "psychosocial support" OR "emotional support" OR "mental health support" OR "psychological care" OR "psychosocial care" OR "emotional care" OR "psychosocial oncology" OR "psycho-oncology" OR empath* OR reassurance OR "therapeutic communication" OR "supportive communication" OR counseling OR counselling OR psychotherapy OR psychoeducation OR "psychological intervention*" OR "psychosocial intervention*" OR "behavioral intervention*" OR "behavioural intervention*" OR "cognitive behavioral therap*" OR "cognitive behavioural therap*" OR CBT OR mindfulness OR relaxation OR "problem solving" OR "positive psychology" OR "positive affect" OR resilience OR coping OR adjustment OR "psychological adjustment" OR "fear of cancer recurrence" OR "fear of recurrence" OR "fear of progression" OR "cancer worry" OR "illness uncertainty" OR "uncertainty management" OR "cancer-related uncertainty" OR "body image" OR "fertility concern*" OR "sexual health" OR intimacy OR "unmet need*" OR "supportive care need*" OR "supportive care" OR "treatment engagement" OR "patient activation" OR "self-management" OR "self management" OR "self-efficacy" OR "self efficacy" OR "patient education" OR "health education" OR "informational support" OR "information support" OR "survivorship care" OR "palliative care")  OR AB (psycholog* OR psychosocial OR psycho-oncolog* OR psychooncolog* OR "mental health" OR "mental wellbeing" OR "mental well-being" OR "psychological distress" OR "emotional distress" OR distress OR anxiety OR anxious OR depression OR depressive OR stress OR loneliness OR "emotional wellbeing" OR "emotional well-being" OR wellbeing OR well-being OR "quality of life" OR "psychological support" OR "psychosocial support" OR "emotional support" OR "mental health support" OR "psychological care" OR "psychosocial care" OR "emotional care" OR "psychosocial oncology" OR "psycho-oncology" OR empath* OR reassurance OR "therapeutic communication" OR "supportive communication" OR counseling OR counselling OR psychotherapy OR psychoeducation OR "psychological intervention*" OR "psychosocial intervention*" OR "behavioral intervention*" OR "behavioural intervention*" OR "cognitive behavioral therap*" OR "cognitive behavioural therap*" OR CBT OR mindfulness OR relaxation OR "problem solving" OR "positive psychology" OR "positive affect" OR resilience OR coping OR adjustment OR "psychological adjustment" OR "fear of cancer recurrence" OR "fear of recurrence" OR "fear of progression" OR "cancer worry" OR "illness uncertainty" OR "uncertainty management" OR "cancer-related uncertainty" OR "body image" OR "fertility concern*" OR "sexual health" OR intimacy OR "unmet need*" OR "supportive care need*" OR "supportive care" OR "treatment engagement" OR "patient activation" OR "self-management" OR "self management" OR "self-efficacy" OR "self efficacy" OR "patient education" OR "health education" OR "informational support" OR "information support" OR "survivorship care" OR "palliative care") |
| S4 **Primary focused search**  **(122)** | S1 AND S2 AND S3 |

APA PsycINFO via EBSCOhost:2026/05/06

| Search set | Search terms |
| --- | --- |
| **S1 AI-based conversational agents**  **(10,115)** | TI (chatbot* OR "chat bot*" OR chatterbot* OR "AI bot*" OR "chat robot*" OR "conversational agent*" OR "conversational AI" OR "conversational system*" OR "dialogue system*" OR "dialog system*" OR "virtual agent*" OR "digital agent*" OR "digital assistant*" OR "virtual assistant*" OR "intelligent assistant*" OR "voice assistant*" OR "relational agent*" OR "embodied conversational agent*" OR "social robot*" OR "social bot*" OR "AI companion*" OR "virtual coach*" OR "digital coach*" OR "e-coach*" OR "nurse avatar*" OR "nursing avatar*" OR "virtual avatar*" OR "conversational avatar*" OR "large language model*" OR "language model*" OR "foundation model*" OR LLM OR LLMs OR "generative AI" OR "generative artificial intelligence" OR "generative pre-trained transformer*" OR "generative pretrained transformer*" OR "AI agent*" OR "artificial intelligence agent*" OR "agentic AI" OR ChatGPT OR "GPT-3*" OR "GPT-4*" OR "GPT-5*" OR OpenAI OR "Bing Chat" OR "Google Bard" OR "Bard AI" OR "Google Gemini" OR "Gemini AI" OR Claude OR "Claude AI" OR "Microsoft Copilot" OR "question answering" OR "question-answering" OR "question answer* system*" OR Vivibot)  OR AB (chatbot* OR "chat bot*" OR chatterbot* OR "AI bot*" OR "chat robot*" OR "conversational agent*" OR "conversational AI" OR "conversational system*" OR "dialogue system*" OR "dialog system*" OR "virtual agent*" OR "digital agent*" OR "digital assistant*" OR "virtual assistant*" OR "intelligent assistant*" OR "voice assistant*" OR "relational agent*" OR "embodied conversational agent*" OR "social robot*" OR "social bot*" OR "AI companion*" OR "virtual coach*" OR "digital coach*" OR "e-coach*" OR "nurse avatar*" OR "nursing avatar*" OR "virtual avatar*" OR "conversational avatar*" OR "large language model*" OR "language model*" OR "foundation model*" OR LLM OR LLMs OR "generative AI" OR "generative artificial intelligence" OR "generative pre-trained transformer*" OR "generative pretrained transformer*" OR "AI agent*" OR "artificial intelligence agent*" OR "agentic AI" OR ChatGPT OR "GPT-3*" OR "GPT-4*" OR "GPT-5*" OR OpenAI OR "Bing Chat" OR "Google Bard" OR "Bard AI" OR "Google Gemini" OR "Gemini AI" OR Claude OR "Claude AI" OR "Microsoft Copilot" OR "question answering" OR "question-answering" OR "question answer* system*" OR Vivibot) |
| **S2 Cancer**  **(110,451)** | DE "Neoplasms"  OR DE "Cancer"  OR DE "Cancer Survivors"  OR TI (cancer* OR neoplasm* OR neoplastic OR neoplasia* OR tumor* OR tumour* OR carcinoma* OR malignan* OR oncolog* OR leukemia* OR leukaemia* OR lymphoma* OR melanoma* OR sarcoma* OR blastoma* OR myeloma* OR "solid tumor*" OR "solid tumour*" OR "hematologic malignan*" OR "haematologic malignan*" OR "cancer survivor*" OR "cancer survivorship")  OR AB (cancer* OR neoplasm* OR neoplastic OR neoplasia* OR tumor* OR tumour* OR carcinoma* OR malignan* OR oncolog* OR leukemia* OR leukaemia* OR lymphoma* OR melanoma* OR sarcoma* OR blastoma* OR myeloma* OR "solid tumor*" OR "solid tumour*" OR "hematologic malignan*" OR "haematologic malignan*" OR "cancer survivor*" OR "cancer survivorship") |
| **S3 Psychological support-related terms**  **(2,111,592)** | DE "Mental Health"  OR DE "Psychotherapy"  OR DE "Cognitive Behavior Therapy"  OR DE "Behavior Therapy"  OR DE "Counseling"  OR DE "Mindfulness"  OR DE "Social Support"  OR DE "Stress"  OR DE "Psychological Stress"  OR DE "Coping Behavior"  OR DE "Adaptation"  OR DE "Anxiety"  OR DE "Depression"  OR DE "Depressive Disorders"  OR DE "Quality of Life"  OR TI (psycholog* OR psychosocial OR psycho-oncolog* OR psychooncolog* OR "mental health" OR "mental wellbeing" OR "mental well-being" OR "psychological distress" OR "emotional distress" OR distress OR anxiety OR anxious OR depression OR depressive OR stress OR loneliness OR "emotional wellbeing" OR "emotional well-being" OR wellbeing OR well-being OR "quality of life" OR "psychological support" OR "psychosocial support" OR "emotional support" OR "mental health support" OR "psychological care" OR "psychosocial care" OR "emotional care" OR "psychosocial oncology" OR "psycho-oncology" OR empath* OR reassurance OR "therapeutic communication" OR "supportive communication" OR counseling OR counselling OR psychotherapy OR psychoeducation OR "psychological intervention*" OR "psychosocial intervention*" OR "behavioral intervention*" OR "behavioural intervention*" OR "cognitive behavioral therap*" OR "cognitive behavioural therap*" OR CBT OR mindfulness OR relaxation OR "problem solving" OR "positive psychology" OR "positive affect" OR resilience OR coping OR adjustment OR "psychological adjustment" OR "fear of cancer recurrence" OR "fear of recurrence" OR "fear of progression" OR "cancer worry" OR "illness uncertainty" OR "uncertainty management" OR "cancer-related uncertainty" OR "body image" OR "fertility concern*" OR "sexual health" OR intimacy OR "unmet need*" OR "supportive care need*" OR "supportive care" OR "treatment engagement" OR "patient activation" OR "self-management" OR "self management" OR "self-efficacy" OR "self efficacy" OR "patient education" OR "health education" OR "informational support" OR "information support" OR "survivorship care" OR "palliative care")  OR AB (psycholog* OR psychosocial OR psycho-oncolog* OR psychooncolog* OR "mental health" OR "mental wellbeing" OR "mental well-being" OR "psychological distress" OR "emotional distress" OR distress OR anxiety OR anxious OR depression OR depressive OR stress OR loneliness OR "emotional wellbeing" OR "emotional well-being" OR wellbeing OR well-being OR "quality of life" OR "psychological support" OR "psychosocial support" OR "emotional support" OR "mental health support" OR "psychological care" OR "psychosocial care" OR "emotional care" OR "psychosocial oncology" OR "psycho-oncology" OR empath* OR reassurance OR "therapeutic communication" OR "supportive communication" OR counseling OR counselling OR psychotherapy OR psychoeducation OR "psychological intervention*" OR "psychosocial intervention*" OR "behavioral intervention*" OR "behavioural intervention*" OR "cognitive behavioral therap*" OR "cognitive behavioural therap*" OR CBT OR mindfulness OR relaxation OR "problem solving" OR "positive psychology" OR "positive affect" OR resilience OR coping OR adjustment OR "psychological adjustment" OR "fear of cancer recurrence" OR "fear of recurrence" OR "fear of progression" OR "cancer worry" OR "illness uncertainty" OR "uncertainty management" OR "cancer-related uncertainty" OR "body image" OR "fertility concern*" OR "sexual health" OR intimacy OR "unmet need*" OR "supportive care need*" OR "supportive care" OR "treatment engagement" OR "patient activation" OR "self-management" OR "self management" OR "self-efficacy" OR "self efficacy" OR "patient education" OR "health education" OR "informational support" OR "information support" OR "survivorship care" OR "palliative care") |
| S4 **Primary focused search**  **(50)** | S1 AND S2 AND S3 |

Web of Science Core Collection:2026/05/06

| Search set | Search terms |
| --- | --- |
| **#1 AI-based conversational agents**  **([99,856](https://libdb.csu.edu.cn/https/vpn/225/P7TXE55GPNSXT3LPMNTT6Z5MMF3GT7UBPSTT6Z5P/wos/woscc/summary/363a7b56-2c41-49f3-98ca-bef0af469cee-01b17398a9/relevance/1))** | TS=(  chatbot* OR "chat bot*" OR chatterbot* OR "AI bot*" OR "chat robot*" OR "conversational agent*" OR "conversational AI" OR "conversational system*" OR "dialogue system*" OR "dialog system*" OR "virtual agent*" OR "digital agent*" OR "digital assistant*" OR "virtual assistant*" OR "intelligent assistant*" OR "voice assistant*" OR "relational agent*" OR "embodied conversational agent*" OR "social robot*" OR "social bot*" OR "AI companion*" OR "virtual coach*" OR "digital coach*" OR "e-coach*" OR "nurse avatar*" OR "nursing avatar*" OR "virtual avatar*" OR "conversational avatar*" OR "large language model*" OR "language model*" OR "foundation model*" OR LLM OR LLMs OR "generative AI" OR "generative artificial intelligence" OR "generative pre-trained transformer*" OR "generative pretrained transformer*" OR "AI agent*" OR "artificial intelligence agent*" OR "agentic AI" OR ChatGPT OR "GPT-3*" OR "GPT-4*" OR "GPT-5*" OR OpenAI OR "Bing Chat" OR "Google Bard" OR "Bard AI" OR "Google Gemini" OR "Gemini AI" OR Claude OR "Claude AI" OR "Microsoft Copilot" OR "question answering" OR "question-answering" OR "question answer* system*" OR Vivibot  ) |
| **#2 Cancer**  **(5,245,169)** | TS=(  cancer* OR neoplasm* OR neoplastic OR neoplasia* OR tumor* OR tumour* OR carcinoma* OR malignan* OR oncolog* OR leukemia* OR leukaemia* OR lymphoma* OR melanoma* OR sarcoma* OR blastoma* OR myeloma* OR "solid tumor*" OR "solid tumour*" OR "hematologic malignan*" OR "haematologic malignan*" OR "cancer survivor*" OR "cancer survivorship"  ) |
| **#3 Psychological support-related terms**  **(6,090,880)** | TS=(  psycholog* OR psychosocial OR psycho-oncolog* OR psychooncolog* OR "mental health" OR "mental wellbeing" OR "mental well-being" OR "psychological distress" OR "emotional distress" OR distress OR anxiety OR anxious OR depression OR depressive OR stress OR loneliness OR "emotional wellbeing" OR "emotional well-being" OR wellbeing OR well-being OR "quality of life" OR "psychological support" OR "psychosocial support" OR "emotional support" OR "mental health support" OR "psychological care" OR "psychosocial care" OR "emotional care" OR "psychosocial oncology" OR "psycho-oncology" OR empath* OR reassurance OR "therapeutic communication" OR "supportive communication" OR counseling OR counselling OR psychotherapy OR psychoeducation OR "psychological intervention*" OR "psychosocial intervention*" OR "behavioral intervention*" OR "behavioural intervention*" OR "cognitive behavioral therap*" OR "cognitive behavioural therap*" OR CBT OR mindfulness OR relaxation OR "problem solving" OR "positive psychology" OR "positive affect" OR resilience OR coping OR adjustment OR "psychological adjustment" OR "fear of cancer recurrence" OR "fear of recurrence" OR "fear of progression" OR "cancer worry" OR "illness uncertainty" OR "uncertainty management" OR "cancer-related uncertainty" OR "body image" OR "fertility concern*" OR "sexual health" OR intimacy OR "unmet need*" OR "supportive care need*" OR "supportive care" OR "treatment engagement" OR "patient activation" OR "self-management" OR "self management" OR "self-efficacy" OR "self efficacy" OR "patient education" OR "health education" OR "informational support" OR "information support" OR "survivorship care" OR "palliative care"  ) |
| #4 **Primary focused search**  **(601)** | #1 AND #2 AND #3 |

Scopus:2026/05/06

| Search set | Search terms |
| --- | --- |
| **#1 AI-based conversational agents**  **(302,330)** | TITLE-ABS-KEY(  chatbot* OR "chat bot" OR "chat bots" OR chatterbot* OR "AI bot" OR "AI bots" OR "chat robot" OR "chat robots" OR "conversational agent" OR "conversational agents" OR "conversational AI" OR "conversational system" OR "conversational systems" OR "dialogue system" OR "dialogue systems" OR "dialog system" OR "dialog systems" OR "virtual agent" OR "virtual agents" OR "digital agent" OR "digital agents" OR "digital assistant" OR "digital assistants" OR "virtual assistant" OR "virtual assistants" OR "intelligent assistant" OR "intelligent assistants" OR "voice assistant" OR "voice assistants" OR "relational agent" OR "relational agents" OR "embodied conversational agent" OR "embodied conversational agents" OR "social robot" OR "social robots" OR "social bot" OR "social bots" OR "AI companion" OR "AI companions" OR "virtual coach" OR "virtual coaches" OR "digital coach" OR "digital coaches" OR "e-coach" OR "e-coaches" OR "ecoach" OR "ecoaches" OR "nurse avatar" OR "nurse avatars" OR "nursing avatar" OR "nursing avatars" OR "virtual avatar" OR "virtual avatars" OR "conversational avatar" OR "conversational avatars" OR "large language model" OR "large language models" OR "language model" OR "language models" OR "foundation model" OR "foundation models" OR LLM OR LLMs OR "generative AI" OR "generative artificial intelligence" OR "generative pre-trained transformer" OR "generative pre-trained transformers" OR "generative pretrained transformer" OR "generative pretrained transformers" OR "AI agent" OR "AI agents" OR "artificial intelligence agent" OR "artificial intelligence agents" OR "agentic AI" OR ChatGPT OR "GPT-3" OR "GPT 3" OR "GPT-3.5" OR "GPT 3.5" OR "GPT-4" OR "GPT 4" OR "GPT-5" OR "GPT 5" OR OpenAI OR "Bing Chat" OR "Google Bard" OR "Bard AI" OR "Google Gemini" OR "Gemini AI" OR Claude OR "Claude AI" OR "Microsoft Copilot" OR "question answering" OR "question-answering" OR "question answering system" OR "question answering systems" OR "question answer system" OR "question answer systems" OR Vivibot  ) |
| **#2 Cancer**  **(7,612,893)** | TITLE-ABS-KEY(  cancer* OR neoplasm* OR neoplastic OR neoplasia* OR tumor* OR tumour* OR carcinoma* OR malignan* OR oncolog* OR leukemia* OR leukaemia* OR lymphoma* OR melanoma* OR sarcoma* OR blastoma* OR myeloma* OR "solid tumor" OR "solid tumors" OR "solid tumour" OR "solid tumours" OR "hematologic malignancy" OR "hematologic malignancies" OR "haematologic malignancy" OR "haematologic malignancies" OR "cancer survivor" OR "cancer survivors" OR "cancer survivorship"  ) |
| **#3 Psychological support-related terms**  **(10,715,011)** | TITLE-ABS-KEY(  psycholog* OR psychosocial OR psycho-oncolog* OR psychooncolog* OR "mental health" OR "mental wellbeing" OR "mental well-being" OR "psychological distress" OR "emotional distress" OR distress OR anxiety OR anxious OR depression OR depressive OR stress OR loneliness OR "emotional wellbeing" OR "emotional well-being" OR wellbeing OR well-being OR "quality of life" OR "psychological support" OR "psychosocial support" OR "emotional support" OR "mental health support" OR "psychological care" OR "psychosocial care" OR "emotional care" OR "psychosocial oncology" OR "psycho-oncology" OR empath* OR reassurance OR "therapeutic communication" OR "supportive communication" OR counseling OR counselling OR psychotherapy OR psychoeducation OR "psychological intervention" OR "psychological interventions" OR "psychosocial intervention" OR "psychosocial interventions" OR "behavioral intervention" OR "behavioral interventions" OR "behavioural intervention" OR "behavioural interventions" OR "cognitive behavioral therapy" OR "cognitive behavioral therapies" OR "cognitive behavioural therapy" OR "cognitive behavioural therapies" OR CBT OR mindfulness OR relaxation OR "problem solving" OR "positive psychology" OR "positive affect" OR resilience OR coping OR adjustment OR "psychological adjustment" OR "fear of cancer recurrence" OR "fear of recurrence" OR "fear of progression" OR "cancer worry" OR "illness uncertainty" OR "uncertainty management" OR "cancer-related uncertainty" OR "body image" OR "fertility concern" OR "fertility concerns" OR "sexual health" OR intimacy OR "unmet need" OR "unmet needs" OR "supportive care need" OR "supportive care needs" OR "supportive care" OR "treatment engagement" OR "patient activation" OR "self-management" OR "self management" OR "self-efficacy" OR "self efficacy" OR "patient education" OR "health education" OR "informational support" OR "information support" OR "survivorship care" OR "palliative care"  ) |
| #4 **Primary focused search**  **(1,203)** | #1 AND #2 AND #3 |

Cochrane CENTRAL:2026/05/06

| Search set | Search terms |
| --- | --- |
| **#1 AI-based conversational agents**  **(2451)** | chatbot*:ti,ab,kw OR "chat bot":ti,ab,kw OR "chat bots":ti,ab,kw OR chatterbot*:ti,ab,kw OR "AI bot":ti,ab,kw OR "AI bots":ti,ab,kw OR "chat robot":ti,ab,kw OR "chat robots":ti,ab,kw OR "conversational agent":ti,ab,kw OR "conversational agents":ti,ab,kw OR "conversational AI":ti,ab,kw OR "conversational system":ti,ab,kw OR "conversational systems":ti,ab,kw OR "dialogue system":ti,ab,kw OR "dialogue systems":ti,ab,kw OR "dialog system":ti,ab,kw OR "dialog systems":ti,ab,kw OR "virtual agent":ti,ab,kw OR "virtual agents":ti,ab,kw OR "digital agent":ti,ab,kw OR "digital agents":ti,ab,kw OR "digital assistant":ti,ab,kw OR "digital assistants":ti,ab,kw OR "virtual assistant":ti,ab,kw OR "virtual assistants":ti,ab,kw OR "intelligent assistant":ti,ab,kw OR "intelligent assistants":ti,ab,kw OR "voice assistant":ti,ab,kw OR "voice assistants":ti,ab,kw OR "relational agent":ti,ab,kw OR "relational agents":ti,ab,kw OR "embodied conversational agent":ti,ab,kw OR "embodied conversational agents":ti,ab,kw OR "social robot":ti,ab,kw OR "social robots":ti,ab,kw OR "social bot":ti,ab,kw OR "social bots":ti,ab,kw OR "AI companion":ti,ab,kw OR "AI companions":ti,ab,kw OR "virtual coach":ti,ab,kw OR "virtual coaches":ti,ab,kw OR "digital coach":ti,ab,kw OR "digital coaches":ti,ab,kw OR "e-coach":ti,ab,kw OR "e-coaches":ti,ab,kw OR ecoach*:ti,ab,kw OR "nurse avatar":ti,ab,kw OR "nurse avatars":ti,ab,kw OR "nursing avatar":ti,ab,kw OR "nursing avatars":ti,ab,kw OR "virtual avatar":ti,ab,kw OR "virtual avatars":ti,ab,kw OR "conversational avatar":ti,ab,kw OR "conversational avatars":ti,ab,kw OR "large language model":ti,ab,kw OR "large language models":ti,ab,kw OR "language model":ti,ab,kw OR "language models":ti,ab,kw OR "foundation model":ti,ab,kw OR "foundation models":ti,ab,kw OR LLM:ti,ab,kw OR LLMs:ti,ab,kw OR "generative AI":ti,ab,kw OR "generative artificial intelligence":ti,ab,kw OR "generative pre-trained transformer":ti,ab,kw OR "generative pre-trained transformers":ti,ab,kw OR "generative pretrained transformer":ti,ab,kw OR "generative pretrained transformers":ti,ab,kw OR "AI agent":ti,ab,kw OR "AI agents":ti,ab,kw OR "artificial intelligence agent":ti,ab,kw OR "artificial intelligence agents":ti,ab,kw OR "agentic AI":ti,ab,kw OR ChatGPT:ti,ab,kw OR "GPT-3":ti,ab,kw OR "GPT 3":ti,ab,kw OR "GPT-3.5":ti,ab,kw OR "GPT 3.5":ti,ab,kw OR "GPT-4":ti,ab,kw OR "GPT 4":ti,ab,kw OR "GPT-5":ti,ab,kw OR "GPT 5":ti,ab,kw OR OpenAI:ti,ab,kw OR "Bing Chat":ti,ab,kw OR "Google Bard":ti,ab,kw OR "Bard AI":ti,ab,kw OR "Google Gemini":ti,ab,kw OR "Gemini AI":ti,ab,kw OR Claude:ti,ab,kw OR "Claude AI":ti,ab,kw OR "Microsoft Copilot":ti,ab,kw OR "question answering":ti,ab,kw OR "question-answering":ti,ab,kw OR "question answering system":ti,ab,kw OR "question answering systems":ti,ab,kw OR "question answer system":ti,ab,kw OR "question answer systems":ti,ab,kw OR Vivibot:ti,ab,kw |
| **#2 Cancer**  **(329809)** | cancer*:ti,ab,kw OR neoplasm*:ti,ab,kw OR neoplastic:ti,ab,kw OR neoplasia*:ti,ab,kw OR tumor*:ti,ab,kw OR tumour*:ti,ab,kw OR carcinoma*:ti,ab,kw OR malignan*:ti,ab,kw OR oncolog*:ti,ab,kw OR leukemia*:ti,ab,kw OR leukaemia*:ti,ab,kw OR lymphoma*:ti,ab,kw OR melanoma*:ti,ab,kw OR sarcoma*:ti,ab,kw OR blastoma*:ti,ab,kw OR myeloma*:ti,ab,kw OR "solid tumor":ti,ab,kw OR "solid tumors":ti,ab,kw OR "solid tumour":ti,ab,kw OR "solid tumours":ti,ab,kw OR "hematologic malignancy":ti,ab,kw OR "hematologic malignancies":ti,ab,kw OR "haematologic malignancy":ti,ab,kw OR "haematologic malignancies":ti,ab,kw OR "cancer survivor":ti,ab,kw OR "cancer survivors":ti,ab,kw OR "cancer survivorship":ti,ab,kw |
| **#3 Psychological support-related terms**  **(588182)** | psycholog*:ti,ab,kw OR psychosocial:ti,ab,kw OR psycho-oncolog*:ti,ab,kw OR psychooncolog*:ti,ab,kw OR "mental health":ti,ab,kw OR "mental wellbeing":ti,ab,kw OR "mental well-being":ti,ab,kw OR "psychological distress":ti,ab,kw OR "emotional distress":ti,ab,kw OR distress:ti,ab,kw OR anxiety:ti,ab,kw OR anxious:ti,ab,kw OR depression:ti,ab,kw OR depressive:ti,ab,kw OR stress:ti,ab,kw OR loneliness:ti,ab,kw OR "emotional wellbeing":ti,ab,kw OR "emotional well-being":ti,ab,kw OR wellbeing:ti,ab,kw OR well-being:ti,ab,kw OR "quality of life":ti,ab,kw OR "psychological support":ti,ab,kw OR "psychosocial support":ti,ab,kw OR "emotional support":ti,ab,kw OR "mental health support":ti,ab,kw OR "psychological care":ti,ab,kw OR "psychosocial care":ti,ab,kw OR "emotional care":ti,ab,kw OR "psychosocial oncology":ti,ab,kw OR "psycho-oncology":ti,ab,kw OR empath*:ti,ab,kw OR reassurance:ti,ab,kw OR "therapeutic communication":ti,ab,kw OR "supportive communication":ti,ab,kw OR counseling:ti,ab,kw OR counselling:ti,ab,kw OR psychotherapy:ti,ab,kw OR psychoeducation:ti,ab,kw OR "psychological intervention":ti,ab,kw OR "psychological interventions":ti,ab,kw OR "psychosocial intervention":ti,ab,kw OR "psychosocial interventions":ti,ab,kw OR "behavioral intervention":ti,ab,kw OR "behavioral interventions":ti,ab,kw OR "behavioural intervention":ti,ab,kw OR "behavioural interventions":ti,ab,kw OR "cognitive behavioral therapy":ti,ab,kw OR "cognitive behavioral therapies":ti,ab,kw OR "cognitive behavioural therapy":ti,ab,kw OR "cognitive behavioural therapies":ti,ab,kw OR CBT:ti,ab,kw OR mindfulness:ti,ab,kw OR relaxation:ti,ab,kw OR "problem solving":ti,ab,kw OR "positive psychology":ti,ab,kw OR "positive affect":ti,ab,kw OR resilience:ti,ab,kw OR coping:ti,ab,kw OR adjustment:ti,ab,kw OR "psychological adjustment":ti,ab,kw OR "fear of cancer recurrence":ti,ab,kw OR "fear of recurrence":ti,ab,kw OR "fear of progression":ti,ab,kw OR "cancer worry":ti,ab,kw OR "illness uncertainty":ti,ab,kw OR "uncertainty management":ti,ab,kw OR "cancer-related uncertainty":ti,ab,kw OR "body image":ti,ab,kw OR "fertility concern":ti,ab,kw OR "fertility concerns":ti,ab,kw OR "sexual health":ti,ab,kw OR intimacy:ti,ab,kw OR "unmet need":ti,ab,kw OR "unmet needs":ti,ab,kw OR "supportive care need":ti,ab,kw OR "supportive care needs":ti,ab,kw OR "supportive care":ti,ab,kw OR "treatment engagement":ti,ab,kw OR "patient activation":ti,ab,kw OR "self-management":ti,ab,kw OR "self management":ti,ab,kw OR "self-efficacy":ti,ab,kw OR "self efficacy":ti,ab,kw OR "patient education":ti,ab,kw OR "health education":ti,ab,kw OR "informational support":ti,ab,kw OR "information support":ti,ab,kw OR "survivorship care":ti,ab,kw OR "palliative care":ti,ab,kw |
| #4 **Primary focused search**  **(146)** | #1 AND #2 AND #3 |

IEEE Xplore:2026/05/06	63

(

"All Metadata":chatbot OR "All Metadata":chatbots OR "All Metadata":"chat bot" OR "All Metadata":"chat bots" OR "All Metadata":"conversational agent" OR "All Metadata":"conversational agents" OR "All Metadata":"conversational AI" OR "All Metadata":"virtual agent" OR "All Metadata":"virtual agents" OR "All Metadata":"virtual assistant" OR "All Metadata":"virtual assistants" OR "All Metadata":"embodied conversational agent" OR "All Metadata":"embodied conversational agents" OR "All Metadata":"social robot" OR "All Metadata":"social robots" OR "All Metadata":"large language model" OR "All Metadata":"large language models" OR "All Metadata":LLM OR "All Metadata":LLMs OR "All Metadata":"generative AI" OR "All Metadata":"generative artificial intelligence" OR "All Metadata":ChatGPT OR "All Metadata":"GPT-4" OR "All Metadata":OpenAI OR "All Metadata":Vivibot

)

AND

(

"All Metadata":cancer OR "All Metadata":cancers OR "All Metadata":oncology OR "All Metadata":oncological OR "All Metadata":tumor OR "All Metadata":tumors OR "All Metadata":tumour OR "All Metadata":tumours OR "All Metadata":neoplasm OR "All Metadata":neoplasms OR "All Metadata":carcinoma OR "All Metadata":carcinomas OR "All Metadata":malignancy OR "All Metadata":malignancies OR "All Metadata":leukemia OR "All Metadata":leukaemia OR "All Metadata":lymphoma OR "All Metadata":melanoma OR "All Metadata":sarcoma OR "All Metadata":myeloma OR "All Metadata":"cancer survivor" OR "All Metadata":"cancer survivors" OR "All Metadata":"cancer survivorship"

)

AND

(

"All Metadata":psychological OR "All Metadata":psychosocial OR "All Metadata":"mental health" OR "All Metadata":"psychological distress" OR "All Metadata":"emotional distress" OR "All Metadata":distress OR "All Metadata":anxiety OR "All Metadata":depression OR "All Metadata":depressive OR "All Metadata":stress OR "All Metadata":"emotional support" OR "All Metadata":"psychosocial support" OR "All Metadata":"psychological support" OR "All Metadata":"quality of life" OR "All Metadata":"supportive care" OR "All Metadata":coping OR "All Metadata":psychoeducation OR "All Metadata":psychotherapy OR "All Metadata":counseling OR "All Metadata":counselling OR "All Metadata":mindfulness OR "All Metadata":relaxation OR "All Metadata":"patient education" OR "All Metadata":"survivorship care" OR "All Metadata":"palliative care"

)

ACM Digital Library:2026/05/06	19

Search Within = Abstract

(

chatbot* OR chatterbot* OR "chat bot" OR "chat bots" OR "conversational agent" OR "conversational agents" OR "conversational AI" OR "conversational system" OR "conversational systems" OR "dialogue system" OR "dialogue systems" OR "dialog system" OR "dialog systems" OR "virtual agent" OR "virtual agents" OR "digital assistant" OR "digital assistants" OR "virtual assistant" OR "virtual assistants" OR "voice assistant" OR "voice assistants" OR "relational agent" OR "relational agents" OR "embodied conversational agent" OR "embodied conversational agents" OR "social robot" OR "social robots" OR "virtual coach" OR "virtual coaches" OR "digital coach" OR "digital coaches" OR ecoach* OR "large language model" OR "large language models" OR "language model" OR "language models" OR LLM OR LLMs OR "generative AI" OR "generative artificial intelligence" OR ChatGPT OR GPT OR OpenAI OR "question answering" OR "question answering system" OR "question answering systems" OR Vivibot

)

AND

(

cancer* OR oncolog* OR neoplasm* OR tumor* OR tumour* OR carcinoma* OR malignan* OR leukemia* OR leukaemia* OR lymphoma* OR melanoma* OR sarcoma* OR myeloma* OR "cancer survivor" OR "cancer survivors" OR "cancer survivorship"

)

AND

(

psycholog* OR psychosocial OR psychooncolog* OR "psycho oncology" OR "mental health" OR "mental wellbeing" OR "mental well being" OR "psychological distress" OR "emotional distress" OR distress OR anxiety OR depression OR depressive OR stress OR coping OR psychoeducation OR psychotherapy OR counseling OR counselling OR mindfulness OR relaxation OR resilience OR "quality of life" OR "emotional support" OR "psychosocial support" OR "psychological support" OR "supportive care" OR "patient education" OR "health education" OR "survivorship care" OR "palliative care" OR "treatment engagement" OR "patient activation" OR "self efficacy"

)

CNKI:2026/5/6	56

TKA=('聊天机器人'+'智能聊天机器人'+'对话机器人'+'会话机器人'+'对话式人工智能'+'对话式AI'+'智能问答'+'问答系统'+'虚拟助手'+'语音助手'+'社交机器人'+'虚拟人'+'数字人'+'大语言模型'+'大型语言模型'+'生成式人工智能'+'生成式AI'+'ChatGPT'+'GPT'+'LLM')

and

TKA=('癌症'+'肿瘤'+'恶性肿瘤'+'癌症患者'+'肿瘤患者'+'癌症幸存者'+'肿瘤幸存者'+'癌症生存者'+'肿瘤生存者'+'肿瘤护理'+'肿瘤学'+'肿瘤科')

and

TKA=('心理支持'+'心理护理'+'心理干预'+'心理援助'+'心理痛苦'+'心理困扰'+'情绪支持'+'情绪管理'+'情绪困扰'+'社会心理'+'心理社会'+'社会心理支持'+'焦虑'+'抑郁'+'应对'+'生活质量'+'生存质量'+'支持性照护'+'支持性护理'+'姑息照护'+'患者教育'+'健康教育'+'信息支持'+'治疗参与'+'自我效能'+'复发恐惧'+'进展恐惧'+'疾病不确定感')

Wanfang:2026/5/6 37

主题:("聊天机器人" OR "智能聊天机器人" OR "对话机器人" OR "对话式人工智能" OR "对话式AI" OR "会话机器人" OR "智能问答" OR "问答系统" OR "虚拟助手" OR "智能助手" OR "虚拟人" OR "数字人" OR "大语言模型" OR "生成式人工智能" OR "生成式AI" OR "ChatGPT" OR "GPT" OR "LLM")

AND

主题:("癌症" OR "肿瘤" OR "恶性肿瘤" OR "癌症患者" OR "肿瘤患者" OR "癌症幸存者" OR "肿瘤幸存者" OR "肿瘤护理" OR "肿瘤学" OR "肿瘤科")

AND

主题:("心理支持" OR "心理护理" OR "心理干预" OR "心理痛苦" OR "心理困扰" OR "情绪支持" OR "情绪管理" OR "焦虑" OR "抑郁" OR "应对" OR "生活质量" OR "生存质量" OR "支持性照护" OR "支持性护理" OR "健康教育" OR "患者教育" OR "信息支持" OR "治疗参与" OR "自我效能")

SinoMed:2026/5/6 4

核心字段

聊天机器人 OR 智能聊天机器人 OR 对话机器人 OR 会话机器人 OR 对话式人工智能 OR 对话式AI OR 智能问答 OR 问答系统 OR 虚拟助手 OR 智能助手 OR 语音助手 OR 社交机器人 OR 虚拟人 OR 数字人 OR 大语言模型 OR 大型语言模型 OR 生成式人工智能 OR 生成式AI OR ChatGPT OR GPT OR LLM

AND

癌症 OR 肿瘤 OR 恶性肿瘤 OR 癌症患者 OR 肿瘤患者 OR 癌症幸存者 OR 肿瘤幸存者 OR 癌症生存者 OR 肿瘤生存者 OR 肿瘤护理 OR 肿瘤学 OR 肿瘤科

AND

心理支持 OR 心理护理 OR 心理干预 OR 心理痛苦 OR 心理困扰 OR 情绪支持 OR 情绪管理 OR 情绪困扰 OR 社会心理 OR 心理社会 OR 焦虑 OR 抑郁 OR 应对 OR 生活质量 OR 生存质量 OR 支持性照护 OR 支持性护理 OR 姑息照护 OR 健康教育 OR 患者教育 OR 信息支持 OR 治疗参与 OR 自我效能 OR 复发恐惧 OR 进展恐惧 OR 疾病不确定感

VIP:2026/5/6	130

(

M=(聊天机器人 OR 智能聊天机器人 OR 对话机器人 OR 会话机器人 OR 对话式人工智能 OR 对话式AI OR 智能问答 OR 问答系统 OR 虚拟助手 OR 智能助手 OR 社交机器人 OR 虚拟人 OR 数字人 OR 大语言模型 OR 生成式人工智能 OR 生成式AI OR ChatGPT)

)

AND

(

M=(癌症 OR 肿瘤 OR 恶性肿瘤 OR 癌症患者 OR 肿瘤患者 OR 肿瘤护理 OR 肿瘤学)

OR

R=(癌症 OR 肿瘤 OR 恶性肿瘤 OR 癌症患者 OR 肿瘤患者 OR 肿瘤护理 OR 肿瘤学)

)

AND

(

M=(心理支持 OR 心理护理 OR 心理干预 OR 心理痛苦 OR 心理困扰 OR 情绪支持 OR 情绪管理 OR 焦虑 OR 抑郁 OR 应对 OR 生活质量 OR 生存质量 OR 支持性照护 OR 支持性护理 OR 姑息照护 OR 复发恐惧 OR 进展恐惧 OR 疾病不确定感)

OR

R=(心理支持 OR 心理护理 OR 心理干预 OR 心理痛苦 OR 心理困扰 OR 情绪支持 OR 情绪管理 OR 焦虑 OR 抑郁 OR 应对 OR 生活质量 OR 生存质量 OR 支持性照护 OR 支持性护理 OR 姑息照护 OR 复发恐惧 OR 进展恐惧 OR 疾病不确定感)

)

<https://clinicaltrials.gov/>：8 （7个排除，1个信息不足）

<https://trialsearch.who.int/> 4 （与检索重复的2个，1个初筛删，未重复的1个信息不全）
