# Supplementary material for: Mapping Technologies, Support Functions, Evaluation Outcomes, and Safety Governance Related to Conversational AI Agents for Psychological Support of Patients With Cancer: Scoping Review
Source: J Med Internet Res. 2026 Oct 19;28:e105599. doi: 10.2196/105599 (PMC13728763; doi:10.2196/105599)
Supplement: Supplementary file 2 [file jmir_v28i1e105599_app2.docx]

Table S1. Characteristics and final functional coding of the 24 included reports

| **Report/project (study unit)** | **Country/region** | **Design** | **Population/cancer context** | **AI system / primary form** | **Final mapped functions by evidence state** | **Evaluation focus** |
| --- | --- | --- | --- | --- | --- | --- |
| R01/P01 (S01)  Akdogan et al. (2025) [1] | Türkiye | Randomized controlled trial | Newly diagnosed patients with solid tumors before chemotherapy | ChatGPT-4.0  LLM/generative AI | D: F1 | Anxiety, depression, and oncologist-rated response appropriateness |
| R02/P02 (S02)  Tsai et al. (2025) [2] | United States | Pilot waitlist randomized controlled trial protocol | Breast cancer survivors with moderate-to-severe insomnia | Cecebot  Rule/knowledge/retrieval text agent | P: F1, F4, F5, F7 | Planned feasibility, acceptability, safety, and exploratory sleep outcomes |
| R03/P03 (S03)  Tawfik et al. (2023) [3] | Egypt | Three-arm randomized controlled trial | Women with breast cancer receiving chemotherapy | ChemoFreeBot  Rule/knowledge/retrieval text agent | D: F1, F4, F7 | Self-care behaviors, symptom burden, and chatbot usability |
| R04/P04 (S04)  Lee et al. (2025) [4] | South Korea | Four-arm randomized controlled trial | Women with breast cancer scheduled for postoperative radiotherapy | Radiotherapy education chatbot  Rule/knowledge/retrieval text agent | D: F1, F7 | Radiotherapy-related anxiety, engagement, and subgroup effects |
| R05/P05 (S05)  van Bindsbergen et al. (2022) [5] | Netherlands | Prospective feasibility study | Children aged 8–12 years receiving anticancer treatment | Hero (NAO6 social robot)  Social robot/embodied agent | D: F1, F7 | Feasibility, engagement, acceptability, and sleep hygiene |
| R06/P06 (S06)  Arem et al. (2020) [6] | United States | Feasibility and prototype development study | Breast cancer survivors with insomnia | Sleep Helper  Voice assistant/smart speaker | D: F1, F4, F5, F7 | Feasibility, acceptability, and privacy concerns |
| R07/P07 (S07)  Alanezi (2025) [7] | Saudi Arabia | Post-intervention qualitative study | Oncology outpatients using ChatGPT for cancer-related questions | ChatGPT-3.5  LLM/generative AI | U: F1, F3, F4, F7 | Patient-perceived benefits, barriers, privacy, personalization, and reliability |
| R08/P08 (S08)  Schläpfer et al. (2026) [8] | Switzerland / German-speaking Europe | Secondary analysis of randomized trial data | Adults with cancer using CanRelax app 2.0 | CanRelax 2.0 / Lumy  Rule/knowledge/retrieval text agent | D: F1, F2, F3, F4, F5, F7 | Working alliance and psychological distress |
| R09/P09 (S09)  Hasei et al. (2025) [9] | Japan | Exploratory multicenter pilot study | Children, adolescents, and young adults with cancer | GPT-4 psychological-support chatbot  LLM/generative AI | D: F1, F2, F3, F4, F5, F6, F7 | Anxiety, stress, treatment motivation, acceptability, and disclosure |
| R10/P10 (S10)  Khairat et al. (2025) [10] | United States | Descriptive phenomenological qualitative study | Breast and prostate cancer survivors | General AI chatbots (no fixed target system)  Concept/perception; no fixed deployed system | U: F1, F2, F3, F6 | Patient perceptions, trust, privacy, empathy, and preference for human care |
| R11/P11 (S11)  Shaban et al. (2025) [11] | Egypt | Randomized controlled trial | Women with breast cancer | AI education chatbot  Rule/knowledge/retrieval text agent | D: F1, F4, F7 | Knowledge, attitudes toward AI, empowerment, and engagement |
| R12/P12 (S12)  Christiansen et al. (2026) [12] | Sweden / Finland | Simulated qualitative exploration | Simulated prostate cancer sexual health scenario among men who have sex with men | ChatGPT, Claude, Copilot, Gemini LLM/generative AI | S: F1, F2, F3, F6, F7 | Inclusiveness, empathy, specificity, cultural sensitivity, and contextual errors |
| R13/P13 (S13)  Groninger et al. (2025) [13] | United States | Mixed-methods feasibility and acceptability study | Adolescent and young adult cancer survivors with chronic insomnia | More SHEEP  Voice assistant/smart speaker | D: F1, F4, F5, F7 | Feasibility, acceptability, usability, and privacy-protective design |
| R14/P14 (S14)  Jafarnia et al. (2026) [14] | United States | AI response-quality evaluation | AYA melanoma survivorship needs derived from patient survey data | ChatGPT 4.0, Copilot, Gemini  LLM/generative AI | S: F1, F2, F3, F7 | Response quality, empathy, readability, and variability |
| R15/P15 (S15)  Hwang et al. (2026) [15] | South Korea | Dataset construction and chatbot pilot | Childhood cancer survivors | KCCSQA chatbot  LLM/generative AI | D: F1, F2, F3, F7 | Expert-rated response quality, hallucination detection, satisfaction, and reuse intention |
| R16/P16 (S16)  Hou et al. (2025) [16] | Australia | Co-design study | Women after cancer treatment and cancer nurse specialists | Conceptual CA mediator roles  Concept/perception; no fixed deployed system | A: F1, F2, F3, F6, F7 | Preferred mediator roles, role boundaries, empathy concerns, and design principles |
| R17/P17 (S17a; S17b)  Liu et al. (2026) [17] | China | One report containing 2 multicenter randomized subtrials | Cancer patients in radiology appointment, preparation, and consultation settings | REC  LLM/generative AI | D: F1, F2, F3, F7 | Dialogue quality, patient satisfaction, burnout among radiology health care professionals, image quality, and empathy limitations |
| R18/P18 (S18)  Schmitz et al. (2023) [18] | United States | Partial crossover randomized trial | Women with metastatic breast cancer | Nurse AMIE  Voice assistant/smart speaker | D: F1, F2, F3, F4, F5, F7 | Feasibility, acceptability, satisfaction, symptoms, quality of life, and function |
| R19/P19 (S19)  Leung et al. (2025) [19] | Canada | Mixed-methods pre–post study | Patients with HR+/HER2− metastatic breast cancer | AIPL  Rule/knowledge/retrieval text agent | D: F1, F2, F7 | Patient activation, usability, user experience, and unmet information needs |
| R20/P20 (S20)  Hanai et al. (2024) [20] | Japan | Generative AI experiment | Hypothetical cancer survivor sexual health questions | ChatGPT-3.5 and DocsBot  LLM/generative AI | S: F1, F2, F6 | Response content, semantic similarity, sentiment, and non-pharmacological recommendation bias |
| R21/P21 (S21)  van Bussel et al. (2022) [21] | Netherlands | Sequential exploratory mixed-methods study | Former and current cancer patients and physicians | Conceptual virtual assistant  Concept/perception; no fixed deployed system | A: F1, F2, F3, F7 | Acceptance factors, use intention, trust, and perceived use cases |
| R22/P08 (S08)  Schläpfer et al. (2024) [22] | Switzerland / Germany / Austria | Exploratory analysis of RCT use data and self-reports | Adults diagnosed with cancer within the previous 5 years | CanRelax 2.0 / Lumy  Rule/knowledge/retrieval text agent | D: F1, F2, F3, F4, F5, F7 | Engagement, adherence, user feedback, self-efficacy, and target-behavior practice |
| R23/P23 (S23a; S23b)  Seo et al. (2025) [23] | United States / South Korea | Two-study co-design and expert evaluation | Children with cancer, parents, and pediatric care experts | ARCH  LLM/generative AI | A: F1, F2, F3, F6, F7 | Expected roles, communication needs, design considerations, and safety protocols |
| R24/P24 (S24)  Cavalieri et al. (2023) [24] | International / Europe | Multicentre randomized trial protocol | Head and neck cancer survivors after curative treatment | BD4QoL / BiDi chatbot  Multimodal monitoring platform + chatbot | P: F1, F2, F4, F5, F7 | Planned QoL deterioration, self-efficacy, usability, trust, and health technology assessment |

*Note. F1=information/education; F2=navigation/resource recommendation; F3=emotional support; F4=motivation/patient activation; F5=structured psychological/behavioral intervention; F6=disclosure/communication facilitation; F7=symptom/self-management support. D=delivered; P=planned in a protocol; S=simulated; A=anticipated/co-designed; U=participant-reported use of a nonfixed general-purpose system. Functions are non-mutually exclusive. Outcome measurement alone was not used to infer a support function.*

Table S2. Project-level system, function, theory, and human-oversight mapping (n=23 projects)

| **Project / linked evidence units** | **AI system/project** | **Primary form and system boundary** | **Theory/foundation** | **Final mapped functions by evidence state** | **Reported human roles and activities** |
| --- | --- | --- | --- | --- | --- |
| P01  Reports: R01  Studies: S01 | ChatGPT-4.0 | LLM/generative AI  General-purpose LLM used under a study-defined cancer-support workflow | No explicit theoretical basis reported | D: F1 | Role(s): Oncologist/physician  Function(s): Response review; accuracy validation |
| P02  Reports: R02  Studies: S02 | Cecebot | Rule/knowledge/retrieval text agent  Purpose-built rule-based SMS conversational agent | Therapeutic model/components without explicit theory-guided framework | P: F1, F4, F5, F7 | Role(s): NR  Function(s): NR |
| P03  Reports: R03  Studies: S03 | ChemoFreeBot | Rule/knowledge/retrieval text agent  Purpose-built knowledge-based chatbot | Explicit theoretical/conceptual framework | D: F1, F4, F7 | Role(s): Nurse; researcher  Function(s): Intervention/content development; delivery of comparator education. Comparator education is not chatbot oversight. |
| P04  Reports: R04  Studies: S04 | Radiotherapy education chatbot | Rule/knowledge/retrieval text agent  Purpose-built rule/decision-tree chatbot with ML intent recognition | No explicit theoretical basis reported | D: F1, F7 | Role(s): Clinical/research team (discipline incompletely specified)  Function(s): Content preparation/appropriateness checking (unclear detail) |
| P05  Reports: R05  Studies: S05 | Hero (NAO6 social robot) | Social robot/embodied agent  Purpose-built embodied/social robot | Therapeutic model/components without explicit theory-guided framework | D: F1, F7 | Role(s): Research/technical team; professional role unspecified  Function(s): Session operation; content development |
| P06  Reports: R06  Studies: S06 | Sleep Helper | Voice assistant/smart speaker  Purpose-built Alexa voice-assistant prototype | Therapeutic model/components without explicit theory-guided framework | D: F1, F4, F5, F7 | Role(s): Research team  Function(s): Prototype development; qualitative coding/review. Qualitative data coding is a research activity, not system oversight. |
| P07  Reports: R07  Studies: S07 | ChatGPT-3.5 | LLM/generative AI  General-purpose LLM used independently by participants within a study period | No explicit theoretical basis reported | U: F1, F3, F4, F7 | Role(s): Research team; mental-health/eHealth professors  Function(s): Study materials/interview-guide consultation only. This consultation does not establish oversight of chatbot content or use. |
| P08  Reports: R08; R22  Studies: S08 | CanRelax 2.0 / Lumy | Rule/knowledge/retrieval text agent  Purpose-built rule-based agent within a fully automated mHealth intervention | Explicit theoretical/conceptual framework | D: F1, F2, F3, F4, F5, F7 | Role(s): Relevant experts and people with cancer; roles not fully specified in this report  Function(s): Intervention/content development; iterative co-design |
| P09  Reports: R09 Studies: S09 | GPT-4 psychological-support chatbot | LLM/generative AI  Purpose-built GPT-4 chatbot deployed through LINE | No explicit theoretical basis reported | D: F1, F2, F3, F4, F5, F6, F7 | Role(s): Physicians/mental-health professionals/research team (as explicitly described)  Function(s): Prompt/content review; safety monitoring/referral as described |
| P10  Reports: R10 Studies: S10 | General AI chatbots (no fixed target system) | Concept/perception; no fixed deployed system  Perception/experience study; no single deployed agent | No explicit theoretical basis reported | U: F1, F2, F3, F6 | Role(s): Not applicable as implemented system oversight  Function(s): Not applicable |
| P11  Reports: R11  Studies: S11 | AI education chatbot | Rule/knowledge/retrieval text agent  Purpose-built rule-based NLP chatbot | No explicit theoretical basis reported | D: F1, F4, F7 | Role(s): Research/clinical team; exact profession for chatbot oversight unclear  Function(s): Content development/validation not sufficiently specified |
| P12  Reports: R12  Studies: S12 | ChatGPT, Claude, Copilot, Gemini | LLM/generative AI  Four general-purpose LLMs tested in a simulated cancer-support scenario | Explicit theoretical/conceptual framework | S: F1, F2, F3, F6, F7 | Role(s): Researcher  Function(s): Response elicitation and qualitative interpretation; no clinical response oversight. Response analysis does not establish clinical oversight. |
| P13  Reports: R13  Studies: S13 | More SHEEP | Voice assistant/smart speaker  Purpose-built Alexa/voice-assistant CBT-I prototype | Therapeutic model/components without explicit theory-guided framework | D: F1, F4, F5, F7 | Role(s): Research/technical team; oncologists as referral sources  Function(s): Prototype development; technical monitoring; recruitment referral. Recruitment referral is not crisis escalation. |
| P14  Reports: R14  Studies: S14 | ChatGPT 4.0, Copilot, Gemini | LLM/generative AI  Three general-purpose LLMs evaluated with standardized simulated prompts | No explicit theoretical basis reported | S: F1, F2, F3, F7 | Role(s): Physician reviewers; medical-student reviewers  Function(s): Response-quality review; empathy rating |
| P15  Reports: R15  Studies: S15 | KCCSQA chatbot | LLM/generative AI  Purpose-built GPT-4 Turbo/RAG question-answering system | No explicit theoretical basis reported | D: F1, F2, F3, F7 | Role(s): Pediatric oncologists; social workers; mental-health specialists  Function(s): Expert response evaluation; validation; expert-guided regeneration |
| P16  Reports: R16  Studies: S16 | Conceptual CA mediator roles | Concept/perception; no fixed deployed system  Co-designed conceptual agent; ChatGPT used only for familiarization | Explicit theoretical/conceptual framework | A: F1, F2, F3, F6, F7 | Role(s): Cancer nurse specialists  Function(s): Co-design; intervention/content concept development; prospective mediation role |
| P17  Reports: R17  Studies: S17a; S17b | REC | LLM/generative AI  Purpose-built fine-tuned DeepSeek R1 clinical chatbot | No explicit psychosocial theoretical basis reported | D: F1, F2, F3, F7 | Role(s): Nurses; radiologic technologists; radiologists; medical-background evaluators  Function(s): Training-data creation; response validation; human-machine collaboration; alert/escalation |
| P18  Reports: R18  Studies: S18 | Nurse AMIE | Voice assistant/smart speaker  Purpose-built Alexa voice assistant | Therapeutic model/components without explicit theory-guided framework | D: F1, F2, F3, F4, F5, F7 | Role(s): Medical oncologists; study/research team  Function(s): Eligibility confirmation; weekly well-being/symptom checks; technical support; clinical escalation |
| P19  Reports: R19  Studies: S19 | AIPL | Rule/knowledge/retrieval text agent  Purpose-built ML-supported patient-librarian chatbot; not an LLM in evaluated version | No explicit theoretical basis reported | D: F1, F2, F7 | Role(s): Patient-education librarians; content experts; clinical oncology team  Function(s): Content curation/annotation; content validation |
| P20  Reports: R20  Studies: S20 | ChatGPT-3.5 and DocsBot | LLM/generative AI  General-purpose LLM plus guideline-grounded configured chatbot | No explicit theoretical basis reported | S: F1, F2, F6 | Role(s): Research team  Function(s): Prompting and response analysis; no explicit clinical oversight. Prompting and analysis do not establish clinical oversight. |
| P21  Reports: R21  Studies: S21 | Conceptual virtual assistant | Concept/perception; no fixed deployed system  Acceptance study using an exemplary video/concept, not a deployed system | Explicit theoretical/conceptual framework | A: F1, F2, F3, F7 | Role(s): Physicians participated as stakeholders  Function(s): Acceptance elicitation; prospective endorsement/role-boundary input. Stakeholder consultation does not establish oversight during deployment. |
| P23  Reports: R23  Studies: S23a; S23b | ARCH | LLM/generative AI  Purpose-built GPT-3.5-turbo prototype with Child Bot and Expert Bot | No explicit theoretical basis reported | A: F1, F2, F3, F6, F7 | Role(s): Psychologists; therapists; social workers; research team  Function(s): Co-design; expert response review/refinement; safety protocol |
| P24  Reports: R24  Studies: S24 | BD4QoL / BiDi chatbot | Multimodal monitoring platform + chatbot  Multimodal AI monitoring platform with e-coaching chatbot | Therapeutic model/components without explicit theory-guided framework | P: F1, F2, F4, F5, F7 | Role(s): Physicians; nurses; clinical investigators  Function(s): Dashboard monitoring; alert review; counseling/physician contact; clinical supervision |

*R08 and R22 share study unit S08 and project P08. R17 and R23 each contribute two study units: 24 reports − 1 shared unit + 2 additional substudies = 25 study units. Project identifiers are retained from the source map, so P22 is not assigned. NR means not reported. Research participation, comparator delivery, recruitment, and interview-guide consultation are distinguished from oversight of AI content or use. Oversight descriptions do not imply real-time clinical monitoring.*

Table S3. Complete descriptive frequencies

| **Characteristic** | **n/N (%)** |
| --- | --- |
| **Publication year (reports; N=24)** | |
| 2020 | 1/24 (4.2%) |
| 2022 | 2/24 (8.3%) |
| 2023 | 3/24 (12.5%) |
| 2024 | 2/24 (8.3%) |
| 2025 | 11/24 (45.8%) |
| 2026 | 5/24 (20.8%) |
| **Country or region (reports; N=24)** | |
| United States | 6/24 (25.0%) |
| Egypt | 2/24 (8.3%) |
| South Korea | 2/24 (8.3%) |
| Netherlands | 2/24 (8.3%) |
| Japan | 2/24 (8.3%) |
| Switzerland/Germany/Austria or German-speaking Europe | 2/24 (8.3%) |
| Other single-country or multi-country settings | 8/24 (33.3%) |
| **Study design (reports; N=24)** | |
| Completed randomized or crossover trial | 6/24 (25.0%) |
| Secondary, process, or engagement analysis derived from trial data | 2/24 (8.3%) |
| Non-randomized feasibility, pilot, pre–post, or mixed-methods user evaluation study | 6/24 (25.0%) |
| Qualitative, co-design, or acceptance study | 4/24 (16.7%) |
| AI response-quality, simulated, or experimental evaluation | 3/24 (12.5%) |
| Dataset/system development with small pilot evaluation | 1/24 (4.2%) |
| Study protocol | 2/24 (8.3%) |
| **Evidence-unit structure (all included evidence)** | |
| Included reports/articles | 24 |
| Operational study units | 25 |
| Underlying AI projects/systems | 23 |
| **Cancer context (reports; N=24)** | |
| Breast cancer or metastatic breast cancer | 7/24 (29.2%) |
| Heterogeneous adult cancer, solid tumors, or pan-cancer populations | 6/24 (25.0%) |
| Pediatric, childhood, or adolescent and young adult cancer | 5/24 (20.8%) |
| Other specific or mixed cancer contexts | 6/24 (25.0%) |
| **Primary AI system form (projects; N=23)** | |
| LLM/generative AI | 9/23 (39.1%) |
| Rule/knowledge/retrieval text agent | 6/23 (26.1%) |
| Voice assistant/smart speaker | 3/23 (13.0%) |
| Social robot/embodied agent | 1/23 (4.3%) |
| Concept/perception; no fixed deployed system | 3/23 (13.0%) |
| Multimodal monitoring platform + chatbot | 1/23 (4.3%) |
| **Theory/foundation (reports; N=24; mutually exclusive)** | |
| No explicit theoretical basis reported | 12/24 (50.0%) |
| Therapeutic model/components without explicit theory-guided framework | 6/24 (25.0%) |
| Explicit theoretical/conceptual framework | 6/24 (25.0%) |
| **Psychological support function (any mapped evidence) (projects; N=23; non-mutually exclusive)** | |
| F1 Information/education | 23/23 (100.0%) |
| F2 Navigation/resource recommendation | 14/23 (60.9%) |
| F3 Emotional support | 12/23 (52.2%) |
| F4 Motivation/patient activation | 10/23 (43.5%) |
| F5 Structured psychological/behavioral intervention | 7/23 (30.4%) |
| F6 Disclosure/communication facilitation | 6/23 (26.1%) |
| F7 Symptom/self-management support | 20/23 (87.0%) |
| **Psychological support function (delivered-only sensitivity view) (projects; N=23; non-mutually exclusive)** | |
| F1 Information/education | 13/23 (56.5%) |
| F2 Navigation/resource recommendation | 6/23 (26.1%) |
| F3 Emotional support | 5/23 (21.7%) |
| F4 Motivation/patient activation | 7/23 (30.4%) |
| F5 Structured psychological/behavioral intervention | 5/23 (21.7%) |
| F6 Disclosure/communication facilitation | 1/23 (4.3%) |
| F7 Symptom/self-management support | 12/23 (52.2%) |
| **Outcome/evaluation domain (reports; N=24; non-mutually exclusive)** | |
| Feasibility | 6/24 (25.0%) |
| Usability | 8/24 (33.3%) |
| Acceptability/satisfaction/UX | 17/24 (70.8%) |
| Engagement/use/adherence | 13/24 (54.2%) |
| Psychological/psychosocial clinical | 8/24 (33.3%) |
| Knowledge/attitude/self-efficacy | 8/24 (33.3%) |
| Behavioral/self-management behavior | 9/24 (37.5%) |
| Symptom/sleep/QoL | 6/24 (25.0%) |
| AI response quality/factuality | 7/24 (29.2%) |
| Outcomes related to health care professionals or clinical workflows | 5/24 (20.8%) |
| Safety/adverse-event evaluation | 4/24 (16.7%) |
| **Population/equity reporting (reports; N=24)** | |
| Pediatric/AYA-specific | 6/24 (25.0%) |
| Adults ≥65 explicitly represented | 7/24 (29.2%) |
| Advanced/metastatic explicitly represented | 4/24 (16.7%) |
| Sex/gender reported or specified | 21/24 (87.5%) |
| Race/ethnicity reported | 4/24 (16.7%) |
| Education/occupation/income variable | 14/24 (58.3%) |
| Participant-level rurality | 0/24 (0.0%) |
| Language eligibility/context explicit | 16/24 (66.7%) |
| Digital access/literacy explicit | 11/24 (45.8%) |
| Disease stage explicit | 13/24 (54.2%) |
| **Safety/governance reporting (reports; N=24; explicit reporting only)** | |
| Ethics procedure | 22/24 (91.7%) |
| Human/expert role or oversight-related function | 15/24 (62.5%) |
| Privacy/data protection | 13/24 (54.2%) |
| Accuracy/hallucination control | 12/24 (50.0%) |
| Crisis/escalation/referral | 8/24 (33.3%) |
| Explicit user-facing AI identity | 3/24 (12.5%) |
| Explicit role boundary | 6/24 (25.0%) |
| Explicit non-replacement statement | 5/24 (20.8%) |

Values are n/N (%), with reports (N=24) or projects (N=23) identified in each section. Support, outcome, population, and governance categories may overlap. Counts describe reporting, and do not establish effectiveness or safety.

References

1. Akdogan O, Uyar GC, Yesilbas E, et al. Effect of a ChatGPT-based digital counseling intervention on anxiety and depression in patients with cancer: a prospective, randomized trial. Eur J Cancer. 2025;221:115408. doi:10.1016/j.ejca.2025.115408
2. Tsai CS, Szewczyk W, Drerup M, et al. A personalized, texting-based conversational agent to address sleep disturbance in individuals who have survived breast cancer: protocol for a pilot waitlist randomized controlled trial. JMIR Res Protoc. 2025;14:e62712. doi:10.2196/62712
3. Tawfik E, Ghallab E, Moustafa A. A nurse versus a chatbot ‒ the effect of an empowerment program on chemotherapy-related side effects and the self-care behaviors of women living with breast cancer: a randomized controlled trial. BMC Nurs. 2023;22(1):102. doi:10.1186/s12912-023-01243-7
4. Lee J, Byun HK, Kim YT, Shin J, Kim YB. A study on breast cancer patient care using chatbot and video education for radiation therapy: a randomized controlled trial. Int J Radiat Oncol Biol Phys. 2025;122(1):84-92. doi:10.1016/j.ijrobp.2024.12.012
5. van Bindsbergen KLA, van der Hoek H, van Gorp M, et al. Interactive education on sleep hygiene with a social robot at a pediatric oncology outpatient clinic: feasibility, experiences, and preliminary effectiveness. Cancers (Basel). 2022;14(15):3792. doi:10.3390/cancers14153792
6. Arem H, Scott R, Greenberg D, Kaltman R, Lieberman D, Lewin D. Assessing breast cancer survivors’ perceptions of using voice-activated technology to address insomnia: feasibility study featuring focus groups and in-depth interviews. JMIR Cancer. 2020;6(1):e15859. doi:10.2196/15859
7. Alanezi F. Examining the role of ChatGPT in promoting health behaviors and lifestyle changes among cancer patients. Nutr Health. 2025;31(2):739-748. doi:10.1177/02601060241244563
8. Schläpfer S, Barth J, Heinz P, Held U, Kowatsch T, Witt CM. Association between working alliance and treatment outcomes in a mobile health intervention with a conversational agent (CanRelax). Internet Interv. 2026;44:100929. doi:10.1016/j.invent.2026.100929
9. Hasei J, Hanzawa M, Nagano A, et al. Empowering pediatric, adolescent, and young adult patients with cancer utilizing generative AI chatbots to reduce psychological burden and enhance treatment engagement: a pilot study. Front Digit Health. 2025;7:1543543. doi:10.3389/fdgth.2025.1543543
10. Khairat S, Mehraby H, Masoumi S, et al. Evaluation of cancer survivors’ experience of using AI-based conversational tools: qualitative study. JMIR Cancer. 2025;11:e77390. doi:10.2196/77390
11. Shaban M, Osman YM, Mohamed NA, Shaban MM. Empowering breast cancer clients through AI chatbots: transforming knowledge and attitudes for enhanced nursing care. BMC Nurs. 2025;24(1):994. doi:10.1186/s12912-025-03585-w
12. Christiansen M, Eriksson H, Fagerström L. Generative AI chatbots as digital adjuncts for sexual health information after prostate cancer in men who have sex with men: auto-netnographic study. JMIR Cancer. 2026;12:e81745. doi:10.2196/81745
13. Groninger H, Arem H, Ayangma L, Gong L, Zhou E, Greenberg D. Development of a voice-activated virtual assistant to improve insomnia among young adult cancer survivors: mixed methods feasibility and acceptability study. JMIR Form Res. 2025;9:e64869. doi:10.2196/64869
14. Jafarnia JL, Haff PL, Moore RP, et al. Quality, empathy, and readability of AI chatbot responses to the survivorship needs of adolescents and young adults with melanoma: evaluation study. JMIR Cancer. 2026;12:e84234. doi:10.2196/84234
15. Hwang K, Kim M, Kim M, et al. GPT-empowered question-answer dataset for informative and empathetic support for Korean childhood cancer survivors. Expert Syst Appl. 2026;298:129548. doi:10.1016/j.eswa.2025.129548
16. Hou M, Porter-Steele J, Balaam S, et al. Conversational agents that don't pretend to care: co-designing mediator roles in digital healthcare interventions. In: Proceedings of the 37th Australian Conference on Human-Computer Interaction. Association for Computing Machinery; 2025:94-110. doi:10.1145/3764687.3764692
17. Liu Z, Orouji F, Yang F, et al. A fine-tuned large language model chatbot for multi-scenario radiology cancer care: randomized controlled trial on interaction optimization, emotional support, and provider burnout reduction. J Transl Med. 2026;24:314. doi:10.1186/s12967-026-07738-6
18. Schmitz KH, Kanski B, Gordon B, et al. Technology-based supportive care for metastatic breast cancer patients. Support Care Cancer. 2023;31(7):401. doi:10.1007/s00520-023-07884-3
19. Leung YW, So J, Sidhu A, et al. The extent to which artificial intelligence can help fulfill metastatic breast cancer patient healthcare needs: a mixed-methods study. Curr Oncol. 2025;32(3):145. doi:10.3390/curroncol32030145
20. Hanai A, Ishikawa T, Kawauchi S, Iida Y, Kawakami E. Generative artificial intelligence and non-pharmacological bias: an experimental study on cancer patient sexual health communications. BMJ Health Care Inform. 2024;31(1):e100924. doi:10.1136/bmjhci-2023-100924
21. van Bussel MJP, Odekerken-Schröder GJ, Ou C, Swart RR, Jacobs MJG. Analyzing the determinants to accept a virtual assistant and use cases among cancer patients: a mixed methods study. BMC Health Serv Res. 2022;22(1):890. doi:10.1186/s12913-022-08189-7
22. Schläpfer S, Schneider F, Santhanam P, et al. Engagement with a relaxation and mindfulness mobile app among people with cancer: exploratory analysis of use data and self-reports from a randomized controlled trial. JMIR Cancer. 2024;10:e52386. doi:10.2196/52386
23. Seo W, Kim YH, Kim JE, et al. Enhancing pediatric communication: the role of an AI-driven chatbot in facilitating child-parent-provider interaction. In: Proceedings of the 2025 CHI Conference on Human Factors in Computing Systems. Association for Computing Machinery; 2025:1-16. doi:10.1145/3706598.3713134
24. Cavalieri S, Vener C, LeBlanc M, et al. A multicenter randomized trial for quality of life evaluation by non-invasive intelligent tools during post-curative treatment follow-up for head and neck cancer: clinical study protocol. Front Oncol. 2023;13:1048593. doi:10.3389/fonc.2023.1048593
